# Modification effects of socioeconomic factors on associations between air pollutants and hand, foot, and mouth disease: A multicity time-series study based on heavily polluted areas in the basin area of Sichuan Province, China

**DOI:** 10.1371/journal.pntd.0010896

**Published:** 2022-11-22

**Authors:** Mengyao Li, Yue Ma, Caiying Luo, Qiang Lv, Yaqiong Liu, Tao Zhang, Fei Yin, Tiejun Shui

**Affiliations:** 1 West China School of Public Health and West China Fourth Hospital, Sichuan University, Chengdu, People’s Republic of China; 2 Sichuan Center for Disease Control and Prevention, Chengdu, People’s Republic of China; 3 Yunnan Center for Disease Control and Prevention, Kunming, People’s Republic of China; Universite de Montreal, CANADA

## Abstract

**Background:**

Hand, foot, and mouth disease (HFMD) is a serious threat among children in China. Some studies have found that air pollution is associated with HFMD incidence, but the results showed heterogeneity. In this study, we aimed to explore the heterogeneity of associations between air pollutants and the number of HFMD cases and to identify significant socioeconomic effect modifiers.

**Methods:**

We collected daily surveillance data on HFMD cases in those aged less than 15 years, air pollution variables and meteorological variables from 2015 to 2017 in the basin area of Sichuan Province. We also collected socioeconomic indicator data. We conducted a two-stage multicity time-series analysis. In the first stage, we constructed a distributed lag nonlinear model (DLNM) to obtain cumulative exposure-response curves between each air pollutant and the numbers of HFMD cases for every city. In the second stage, we carried out a multivariable meta-regression to merge the estimations in the first stage and to identify significant socioeconomic effect modifiers.

**Results:**

We found that PM_10_, NO_2_ and O_3_ concentrations were associated with the number of HFMD cases. An inverted V-shaped association between PM_10_ and the number of HFMD cases was observed. The overall NO_2_-HFMD association was a hockey-stick shape. For the relationships of PM_10_, SO_2_, NO_2_, O_3_ and CO with HFMD counts, approximately 58.5%, 48.4%, 51.0%, 55.6% and 52.5% of the heterogeneity could be explained, respectively. The proportion of primary school students, population density, urbanization rate, number of licensed physicians and number of hospital beds explained part of the heterogeneity and modified the relationships.

**Conclusion:**

Our study explored the heterogeneity of associations between air pollutants and HFMD counts. The proportion of primary school students, population density, urbanization rate, number of licensed physicians and number of hospital beds could modify the relationships. The results can serve as a reference for relevant public health decision making.

## Author summary

This study constructed a two-stage multi-city time-series analysis to explore the heterogeneity of air pollutants-HFMD associations and identify significant socioeconomic effect modifiers, which may lead to heterogeneity. In the results, we found that PM_10_, NO_2_ and O_3_ concentrations were associated with HFMD. For each air pollutant-HFMD associations between cities, the heterogeneity was found. The proportion of primary school students, population density, urbanization rate, number of licensed physicians and number of hospital beds explained part of the heterogeneity. The results showed that demographic factors, economic factors, and health resource factors may influence the effect of air pollutants on HFMD, which could further the understanding of the associations between air pollutants and HFMD and could be as evidence for relevant public health decision-making.

## 1. Introduction

Hand, foot, and mouth disease (HFMD), caused by enteroviruses, is a highly prevalent infectious disease mainly occurring in children [1,2]. In recent decades, it has emerged in many Asian countries [1,3–6]. HFMD has a high incidence and imposes a serious disease burden on those in mainland China. From 2008 to 2019, a total of approximately 22.45 million HFMD cases were reported nationwide, ranking first among all notifiable infectious diseases. Although HFMD is usually self-limiting, it may sometimes lead to serious central nervous system and cardiovascular complications and even death [7]. Approximately 75,881 disability-adjusted life years (DALYs) are caused by HFMD annually [8]. Therefore, HFMD is a severe threat among children. However, only inactivated enterovirus A71 vaccines have been developed, and specific therapies are still lacking [9,10]. Thus, nonpharmaceutical interventions are necessary to reduce the risk of HFMD.

Identifying environmental risk factors is important for the implementation of nonpharmaceutical HFMD interventions. Meteorological factors, such as temperature and relative humidity, have been found to be associated with HFMD in many studies [11–14]. In addition, considering persistent air pollution [15], some studies have recently suggested that air pollutants are associated with the HFMD incidence [16–22]. Nonlinear relationships and delayed effects have been shown for most of these associations. However, controversial results have been reported among different studies. For example, a study in Wuhan [16] found that PM_10_ exposure increased the risk of HFMD on lag10-11 according to a generalized additive model (GAM). Another study in Ningbo [19] demonstrated that there was no significant PM_10_-HFMD relationship according to a distributed lag nonlinear model (DLNM). A study in Guangxi [23] stated that the PM_10_-HFMD relationship has a positive linearity according to a principal component regression (PCR). Studies in Chengdu [20] and Shenzhen [21] showed that the cumulative risk curves of PM_10_ and HFMD were inverted "V"-shaped according to the DLNM. Among these studies, heterogeneity of the correlations and the shapes of the association curves can be observed. This heterogeneity may be attributed to different study methods, the modification effects socioeconomic variables and so on [24]. Exploring heterogeneity can reveal the influence of effect modifiers on air pollutant-HFMD associations, which may guide HFMD prevention strategies. However, all previous studies on air pollutant-HFMD associations were conducted in a single city. Thus, the exploration of heterogeneity is still lacking.

To determine the reasons for the heterogeneity and obtain average air pollutant-HFMD associations, a multiregional study with a common statistical method is needed. Therefore, we conducted a multicity study based on 17 cities in Sichuan Province with a two-stage time-series analysis. We selected this area because HFMD in Sichuan Province has always been ranked among the top three notifiable infectious diseases. In addition, the selected 17 cities, located in the basin area, have serious air pollution because of rapid industrialization [25,26]. Moreover, different socioeconomic development levels can be found among cities. Two-stage time-series analyses, consisting of a DLNM and meta-analysis, have been conducted in many epidemiological studies to explore modification effects [13,14,27,28]. The DLNM can characterize nonlinear associations between two variables in the lag-response and exposure-response dimensions [29,30]. The meta-analysis can merge city-specific associations and identify effect modifiers of heterogeneity among cities [27,31].

In this work, we carried out a two-stage time-series analysis to explore the heterogeneity of air pollutant-HFMD case associations and identify socioeconomic effect modifiers in the basin area of Sichuan Province. In the first stage, we obtained cumulative exposure-response curves between air pollutant concentrations and the numbers of HFMD cases for every city. In the second stage, we merged the estimations in the first stage and included the city-specific socioeconomic indicators to identify significant effect modifiers. Our results can serve as reference data for the prevention and control of HFMD.

## 2. Methods

### 2.1 Ethics statement

Our study was approved by the institutional review board of the School of Public Health, Sichuan University. All HFMD surveillance data were collected from Sichuan Center for Disease Control and Prevention. The study methods were carried out in accordance with relevant guidelines and regulations. Our study was constructed at the population level. Therefore, no confidential information was involved in this study and informed consent was not required.

### 2.2 Study area

The 17 selected cities, with a total area of 185,757 km^2^, are located in the basin area of Sichuan Province, China. The study area has a subtropical monsoon climate. The western and southern parts of the basin area are heavily polluted, while the northeastern area is less polluted. The western area has a larger population and more health resources than the northeastern area.

### 2.3 Data collection

Daily HFMD cases in children under the age of 15 in the study areas from January 1, 2015, to December 31, 2017, were collected from Sichuan Center for Disease Control and Prevention. Daily air pollutant data for the same period, including PM_10_ (μg/m^3^), PM_2.5_ (μg/m^3^), SO_2_ (μg/m^3^), NO_2_ (μg/m^3^), CO (mg/m^3^), O_3_ (μg/m^3^) and the air quality index (AQI), were obtained from the China National Environmental Monitoring Center by the arithmetic averaging of hourly data. Daily meteorological data for the same period, including mean temperature (tm, °C), mean relative humidity (humid, %), mean wind velocity (win, m/s), precipitation (rain, mm), and sunshine hours (sun, h), were obtained from the China Meteorological Data Sharing Service System. Missing values for PM_10_, PM_2.5_, SO_2_, NO_2_, O_3_, CO, AQI, tm, humid and win were imputed for 21 days (0.11%), 11 days (0.06%), 19 days (0.10%), 22 days (0.12%), 33 days (0.18%), 16 days (0.09%), 60 days (0.32%), 33 days (0.23%), 2 days (0.01%) and 34 days (0.24%) by linear interpolation. Missing values for sun and rain were imputed for 31 days (0.22%) and 1141 days (8.00%) by “0".

According to previous studies, 4 types of city-specific indicators were included in our study: (1) demographic variables: the population density (people per km^2^), birth rate (‰) and proportion of primary school students (‰); (2) economic variables: gross domestic product (GDP) per person (CNY), GDP increase (%) and urbanization rate (%); (3) health resource variables: the number of licensed physicians (per 1000 population) and number of hospital beds (per 1000 population); and (4) a traffic variable: the total number of passengers (trips). City-specific socioeconomic data were collected from the China City Statistical Yearbooks from 2015 to 2017, and each indicator for each city was calculated by arithmetic averaging of the indicators for three years.

### 2.4 Statistical analysis

A two-stage multicity time-series analysis was carried out to characterize associations between air pollutant concentrations and the number of HFMD cases and identify significant effect modifiers in the study area from 2015 to 2017. First, to obtain the cumulative exposure-response curves of the effects of PM_10_, PM_2.5_, SO_2_, NO_2_, CO, and O_3_ concentrations on the number of HFMD cases, a DLNM was constructed for each city. Then, to obtain overall cumulative exposure-response curves, the estimations in the first stage were merged by multivariable meta-analysis. Finally, each city-specific socioeconomic indicator was included to identify whether the indicator could explain the heterogeneity between cities and evaluate its modification effect.

PM_10_ and PM_2.5_ concentrations were found to be highly correlated (r_s_ = 0.94) by Spearman rank correlation analysis (S1 Fig), which may lead to multicollinearity. Therefore, considering that both particulate matter and PM_10_ were analyzed in several studies, PM_10_, SO_2_, NO_2_, O_3_, and CO were finally included in our study.

#### 2.4.1 First-stage analysis

To measure the cumulative exposure-response curve of the relationship between each air pollutant and the number of HFMD cases for each city, a DLNM was constructed as follows:

$$Y_{t}\sim Quasi-Possion(\mu_{t})$$ (1)

$$\text{log(}\mu_{t})=\alpha +\sum cb\left(P_{it},df,l,df\right)+ns\left(EMA\left(tm_{t},humid_{t},l\right),df\right)+\sum SMA\left(M_{jt},l\right)+ns\left(times,df\right)+\beta Holiday_{t}+\gamma DOW_{t}+\delta A_{t}$$ (2)

where *Y*_*t*_ is the number of HFMD cases on day *t*(*t* = 1,2,…1096); *α* is the intercept; *cb*(·) is the cross-basis function; and *P*_*it*_ is the *i*(*i* = 1,2,…5)th pollutant (*i* = 1,2,…5) on day *t*. Natural cubic splines with 3 degrees of freedom (*df*) were applied to characterize the lag-response relationship between each air pollutant concentration and the number of HFMD cases, and natural cubic splines with 2 knots were applied to characterize the exposure-response relationship. The values of the knots were the 33^th^ and 66^th^ percentiles of each air pollutant concentrations for 17 cities (Knots: PM_10_: 54.3 μg/m^3^, 86.0 μg/m^3^; SO_2_: 11.9 μg/m^3^, 15.7 μg/m^3^; NO_2_: 25.4 μg/m^3^, 33.5 μg/m^3^; O_3_: 45.0 μg/m^3^, 70.5 μg/m^3^; CO: 0.7 mg/m^3^, 0.9 mg/m^3^). Zero to fourteen days was the lag interval for PM_10_, SO_2_, NO_2_, CO, and O_3_. To control for confounding factors, the mean temperature was included by calculating the simple moving weighted averages (SMAs) in the same lag interval as the air pollutants and a natural cubic spline with 3 *df*. *M*_*jt*_ is the *j*(*j* = 1,2,…4)th other meteorological confounder, which was incorporated by calculating the SMAs in the same lag interval as the air pollutants. A natural cubic spline with 8 *df* per calendar year was used to control for seasonal and long-term trends. *Holiday*_*t*_ is a binary variable indicating whether day *t* was a holiday. *DOW*_*t*_ is an indicator of the day of a week. *A*_*t*_ is the autoregressive term to control for autocorrelation. Sensitivity analysis, using the sum of the quasi Akaike Information Criterion (QAIC) of 17 cities as the criterion, was conducted to determine the forms of variables.

#### 2.4.2 Second-stage analysis

The multivariable meta-regression was constructed as follows:

$$\hat{\theta_{q}}\sim N\left(U_{q}\beta ,S_{q}+\Psi \right)$$ (3)

$$U_{q}=I_{\left(k\right)}⊗u_{q}^{T}$$ (4)

where $\hat{\theta_{q}}$ is a *k*-dimensional parameter vector representing the estimate of the association between air pollutants and HFMD in the q^th^ city. *S*_*q*_ is a *k × k* dimensional variance-covariance matrix of $\hat{\theta_{q}}$. *U*_*q*_, a *k × kp* dimensional block-diagonal matrix, is the Kronecker expansion of *p* area-specific indicators *u*_*q*_ = [*u*_1_, *u*_2_…*u*_*p*_]^*T*^. When there is no meta-variable, *U* = *I*_(*k*)_ and *β* denote the overall mean of the parameter vector $\hat{\theta_{q}}$. *S*_*q*_ and *Ψ* are variance-covariance matrices representing within-group variation and between-group variation, respectively.

The multivariable meta-regression with only an intercept and restricted maximum likelihood estimation (REML) were adopted to pool the city-specific estimates from the first stage to obtain the overall cumulative exposure-response associations between the number of HFMD cases and air pollutant concentrations. The heterogeneity of these associations was measured by the Cochran *Q* test and *I*^*2*^ statistic. Then, each city-specific socioeconomic indicator was independently included in the meta-regression to explore whether the indicator could explain the heterogeneity, and the likelihood ratio (LR) test was carried out to identify significant effect modifiers (*P*≤0.05).

All statistical analyses were conducted in R 4.0.3 using the packages *dlnm*, *splines* and *mvmeta*.

## 3. Results

### 3.1. Descriptive analysis

Table 1 shows the summary characteristics of all the variables in our study. A total of 201,035 HFMD cases in individuals aged less than 15 years in 17 cities were reported from 2015 to 2017. The air pollution concentrations were different among cities (S2 Fig). Regarding PM_10_, PM_2.5_, SO_2_, CO and O_3_, the southern cities had heavier pollution. Regarding NO_2_, Chengdu was the most polluted city. Variations were also found among city-specific socioeconomic characteristics (S3 Table). The eastern cities had a higher proportion of primary school students, and the northeastern cities had smaller populations and fewer health resources.

**Table 1:** Descriptions of daily HFMD cases, meteorological factors and air pollutants in the basin area of Sichuan Province from January 1, 2015, to December 31, 2017.

Variable	Mean± SD	Median (IQR)	Range
HFMD (cases)	183.4±100.6	176.0 (104.0, 244.0)	(3.0, 517.0)
Air pollutants			
PM_10_ (μg/m^3^)	79.8±49.1	68.0 (45.0, 103.0)	(6.0, 599.0)
PM_2.5_ (μg/m^3^)	49.9±35.7	40.0 (25.0, 65.0)	(2.0, 370.0)
SO_2_ (μg/m^3^)	14.5±8.5	13.0 (9.0, 18.0)	(1.0, 112)
NO_2_ (μg/m^3^)	30.5±12.7	29.0 (21.0, 38.0)	(4.0, 112.0)
O_3_ (μg/m^3^)	62.3±35.0	56.0 (37.0, 81.0)	(2.0, 242.0)
CO (mg/m^3^)	0.9±0.3	0.8 (0.6, 1.0)	(0.1, 4.0)
AQI	78.1±41.2	67.0 (51.0, 94.0)	(14.0, 399)
Meteorological factors			
Tm (°C)	18.1±7.3	18.6 (11.3, 24.1)	(-2.2, 36.1)
Humid (%)	77.4±12.1	78.0 (69.0, 87.0)	(20.0, 100.0)
Win (m/s)	1.4±0.6	1.3 (1.0, 1.7)	(0.0, 6.4)
Sun (h)	3.3±3.9	1.2 (0.0, 6.6)	(0.0, 13.3)
Rain(mm)	2.8±9.3	0.0 (0.0, 1.3)	(0.0, 266.7)
City-specific indicators			
Population density (people per km^2^)	449.8±249.1	419.1 (269.9, 596.1)	(102.3, 1146.2)
Proportion of students (‰)	62.9±12.9	58.1 (54.1, 73.7)	(49.5, 92.9)
Birth rate (‰)	9.9±0.7	9.8 (9.5, 10.2)	(8.6, 11.3)
GDP per person (CNY)	37,095.6±13,516.0	35676.7(31,106.0, 38,990.3)	(16,543, 79,381.3)
GDP increase (%)	17.0±10.3	19.9 (15.9, 21.1)	(-19.5, 28.6)
Urbanization rate (%)	46.6±7.3	46.6 (42.4, 48.7)	(38.8, 71.3)
Hospital beds(per 1000 population)	2.6±0.4	2.6 (2.3, 2.8)	(1.9, 3.4)
Licensed physicians (per 1000 population)	7.6±1.4	7.5 (6.6, 8)	(6.2, 12.1)
Passengers (trips)	3,055,310.9±251,066.8	234,231.0(165,834.0, 293,041.0)	(911,55.0, 1,167,754.0)

**Fig 1:**
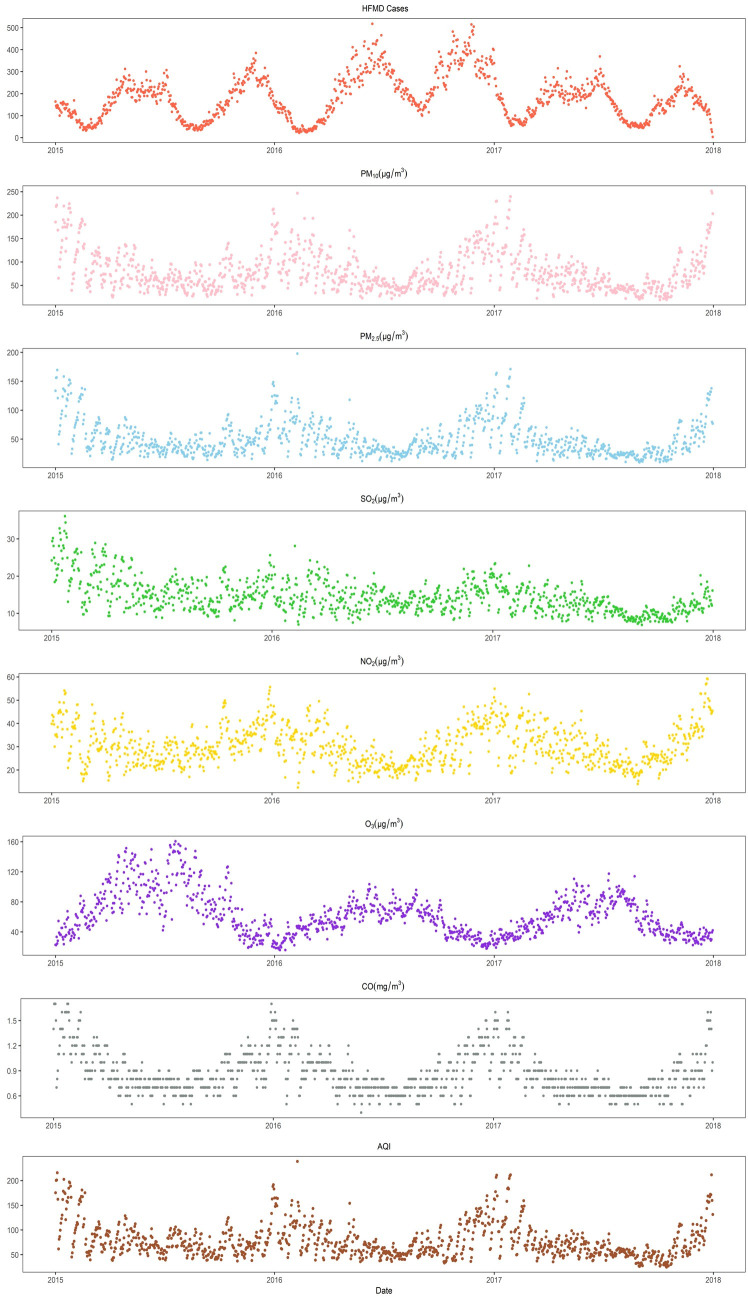
Daily distributions of HFMD cases, air pollutants and the AQI in the basin area of Sichuan Province from 2015 to 2017.

Fig 1 illustrates the seasonal trends of HFMD cases and air pollutants. The number of HFMD cases had two peaks every year, which were concentrated in May-July and November-January. O_3_ had a peak concentration in summer and a lower concentration in winter. In contrast, the other air pollutant concentrations and the AQI were higher in winter and lower in summer.

### 3.2 Overall pooled curves of the air pollutant-HFMD relationship

**Fig 2:**
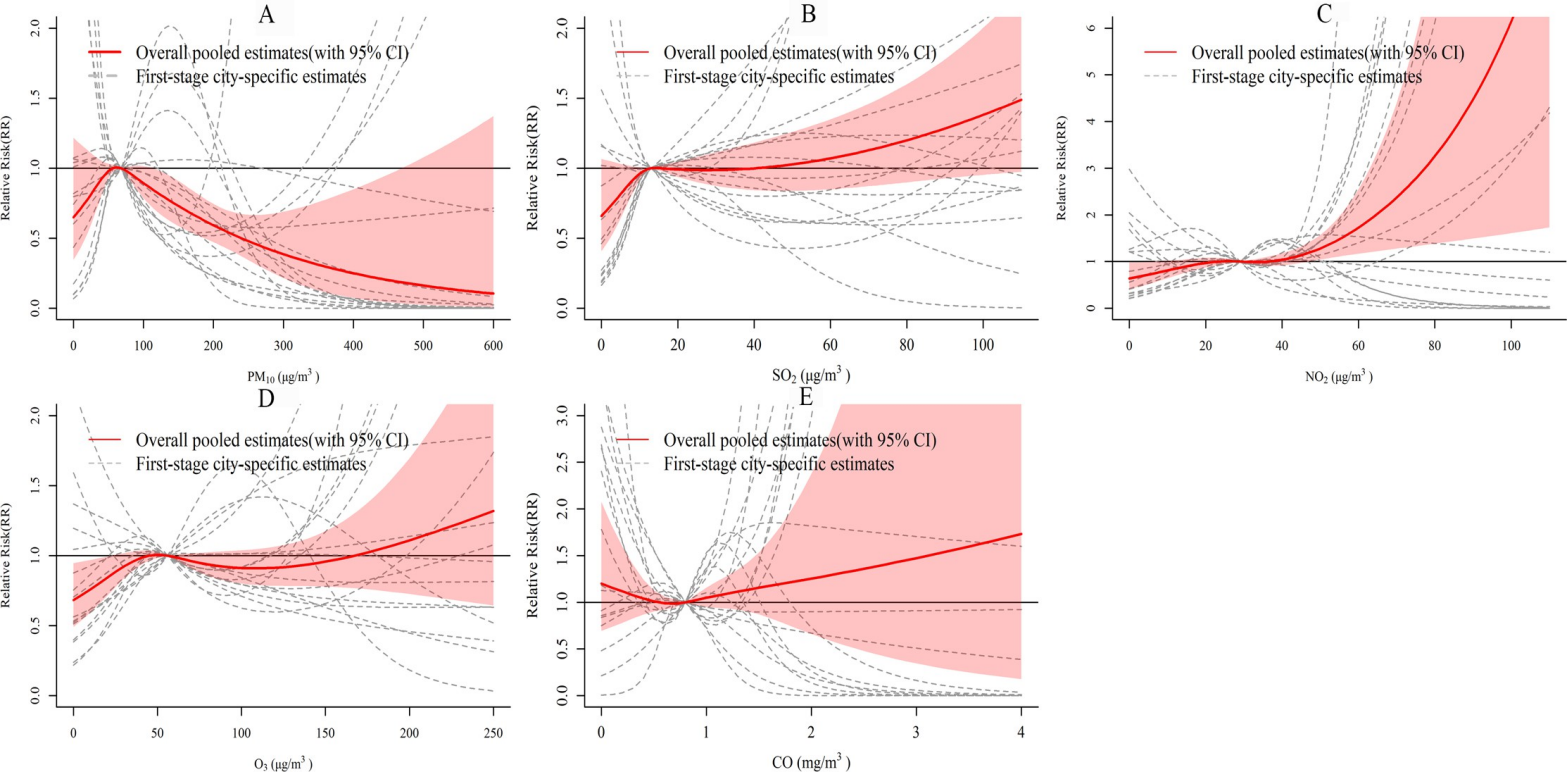
The overall and city-specific cumulative-response curves of the relationships between air pollutant concentrations and the number of HFMD cases. The reference for all estimates was the median of each pollutant.

We merged the city-specific estimates for 17 cities to obtain overall pooled cumulative exposure-response curves and 95% confidence intervals (*CI*s) (Fig 2). Fig 2A indicates that the association between the PM_10_ concentration and the number of HFMD cases has an approximately inverted V-shape. The relative risk (RR) increased between 0–66 μg/m^3^ and peaked before decreasing. The curve of NO_2_ (Fig 2C) was approximately hockey-stick shaped, and the RR reached 4.47 (95% *CI*: 1.49, 13.40) at an NO_2_ concentration of 90 μg/m^3^. For O_3_ (Fig 2D), the RR increased between 0–50 μg/m^3^, decreased slightly between 50–108 μg/m^3^, and then increased with increasing O_3_ concentrations. For SO_2_ (Fig 2B) and CO (Fig 2E), the 95% *CIs* of their curves almost contained 1, meaning that the associations were not statistically significant.

### 3.3 Heterogeneity and effect modifiers of the air pollutant-HFMD relationship

The pooled results showed that the city-specific cumulative exposure-response curves for each pollutant were heterogeneous. Table 2 displays the heterogeneity and significant effect modifiers of the air pollutant-HFMD relationships. The results of the *I*^*2*^ statistic indicated that 58.5%, 48.4%, 51.0%, 55.6% and 52.5% of the variances in the relationships of PM_10_, SO_2_, NO_2_, O_3_ and CO concentrations with the number of HFMD cases could be explained by differences among cities, respectively.

For the effect modifiers, some demographic factors, economic factors and health resource factors could partly explain the heterogeneity of the air pollutant-HFMD relationship. The proportion of primary school students explained 10.9% and 5.9% of the heterogeneity of the associations of SO_2_ and NO_2_ with the number of HFMD cases, respectively. The number of licensed physicians explained 4.2% of the heterogeneity of the association between PM_10_ and the number of HFMD cases. The number of hospital beds, urbanization rate and population density explained 1.5%, 8.2% and 7.4% of the heterogeneity of the relationship between O_3_ and the number of HFMD cases, respectively.

**Table 2:** Heterogeneity and effect modifiers of the relationships between pollutant concentrations and the number of HFMD cases.

Meta-predictor	LR test			Model fit	Cochran Q test			*I* ^*2*^
	*χ*^*2*^ statistic	*df*	*P*	AIC	*χ*^*2*^ statistic	*df*	*P*	(%)
PM_10_								
Intercept only	-	-	-	170.1	115.5	48	<0.001	58.5
Licensed physicians	8.2	3	0.042	168.0	98.4	45	<0.001	54.3
SO_2_								
Intercept only	-	-	-	150.0	93.0	48	<0.001	48.4
Proportion of students	16.5	3	0.001	139.6	72.1	45	0.006	37.5
O_3_								
Intercept only	-	-	-	131.4	108.2	48	<0.001	55.6
Urbanization rate	8.7	3	0.034	128.7	85.6	45	<0.001	47.4
Population density	7.8	3	0.050	129.6	86.9	45	<0.001	48.2
Hospital beds	10.2	3	0.017	127.2	98.0	45	<0.001	54.1
NO_2_								
Intercept only	-	-	-	154.5	98.0	48	<0.001	51.0
Proportion of students	11.6	3	0.009	148.9	82.0	45	0.001	45.1
CO								
Intercept only	-	-	-	151.8	101.1	48	<0.001	52.5

*“-”indicates that the intercept was not tested by the LR test.

**Fig 3:**
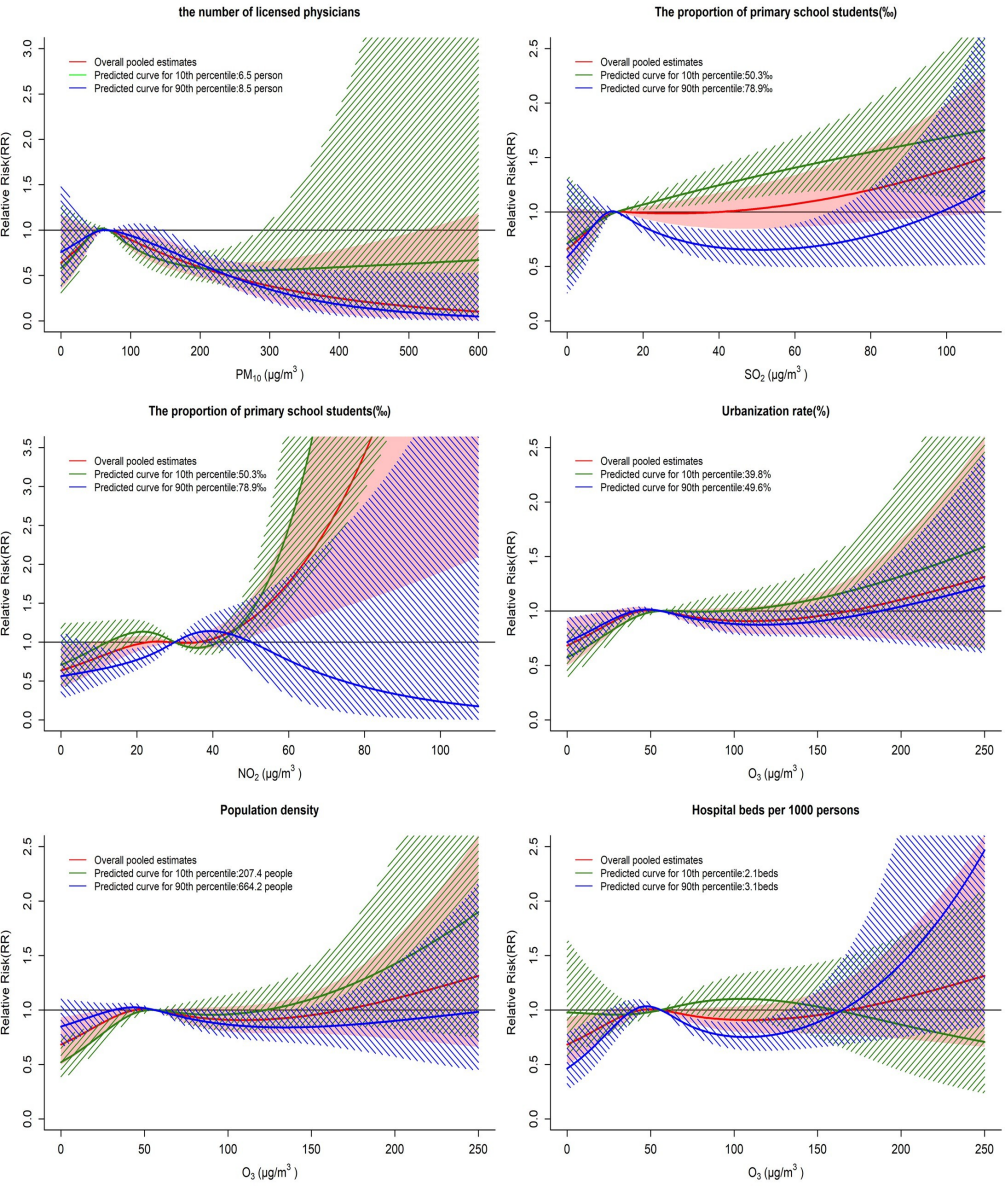
Predicted air pollutant-HFMD associations considering effect modifiers at the 10th and 90th percentiles.

Fig 3 summarizes the estimates of the air pollutant-HFMD associations considering the 10^th^ and 90^th^ percentiles of the effect modifiers. For PM_10_, the small number of licensed physicians weakened the negative association between the high PM_10_ concentration and the number of HFMD cases. For SO_2_, a high proportion of primary school students weakened the positive association with HFMD cases when the concentration exceeded 13 μg/m^3^. For NO_2_, a high proportion of primary school students transformed the positive association with the number of HFMD cases into a nonsignificant negative association when the concentrations exceeded 45 μg/m^3^. For O_3_, low population density and low urbanization rate strengthened the association between O_3_ and the number of HFMD cases. A large number of hospital beds per 1000 persons reduced the RR of HFMD at 0–163 μg/m^3^.

## 4. Discussion

This study explored the relationships between air pollutant concentrations and the number of HFMD cases and identified significant socioeconomic effect modifiers. Our results revealed that PM_10_, NO_2_ and O_3_ concentrations were associated with the number of HFMD cases in a heavily polluted area with a high HFMD incidence and different socioeconomic levels. Moreover, we found heterogeneity for each pollutant in the cumulative exposure-response associations between cities. Regarding the relationships of PM_10_, SO_2_, NO_2_, O_3_ and CO with the number of HFMD cases, approximately 58.5%, 48.4%, 51.0%, 55.6% and 52.5% of the variations could be explained by differences among different cities, respectively. The proportion of primary school students, population density, urbanization rate, number of licensed physicians and number of hospital beds could partly explain the heterogeneity and modify the relationships. The exact mechanisms of the effects of air pollutant concentrations on HFMD cases remain unclear and need further exploration. Our results could help to enhance the understanding of the relationships between air pollutant concentrations and the number of HFMD cases and provide a reference for relevant public health decision-making.

For the relationships between air pollutant concentrations and the number of HFMD cases, we found that the overall association between PM_10_ and the number of HFMD cases had an approximately inverted V-shape. When the PM_10_ concentration was 0–66 μg/m^3^, the correlation was positive. One possible explanation is that PM_10_ may lead to lung function damage and an inflammatory response [32]. Another is that viruses may attach to particles in the air, which may enhance the transmission of the HFMD virus [33–35]. When the PM_10_ concentration exceeded 66 μg/m^3^, we found a negative association, which was consistent with results in Chengdu [20], Shenzhen [21], and Guangxi [23]. This phenomenon may be attributed to early warning measures implemented by the government. “The Sichuan Provincial Heavy Pollution Weather Emergency Plan” [36], published in 2014, provides the following warning: when the daily average AQI exceeds 200 for 3 or more days, kindergartens and primary schools are advised to avoid outdoor activities to effectively reduce children’s exposure to air pollution. In addition, people may opt to wear masks, use air purifiers, and close windows on heavy pollution days as a result of increased awareness of the hazards of particulate matter exposure [37,38], which might also reduce the risk of HFMD.

We found that a low O_3_ concentration had a weakly positive correlation with the number of HFMD cases, which was similar to studies in Guangxi [23] and Ningbo(Gu et al., 2020) and was inconsistent with studies in Guilin(Yu et al., 2019) and Shenzhen(Yan et al., 2019). Some studies have shown that O_3_ exposure could compromise epithelial defenses, increase transmucosal permeability [39], induce oxidative damage to cells and the lining fluids of the airways [40], and cause an acute effect on child lung function [41], which may increase the susceptibility of children to HFMD. However, some other studies have indicated that an appropriate O_3_ concentration could reduce virus production and stimulate cytokine production, which may reduce the risk of EV71 HFMD infection [42]. This may lead to a nonsignificant association with HFMD when the O_3_ concentration exceeds 50 μg/m^3^. Further research is needed to explore the association between O_3_ and HFMD cases and the possible mechanism.

The overall relationship between the NO_2_ concentration and the number of HFMD cases was approximately hockey-stick shaped, and the RR was 4.47 (95% *CI*: 1.49, 13.40) at 90 μg/m^3^, suggesting that high NO_2_ concentrations greatly increase the HFMD risk. A study in Hong Kong found that NO_2_ may cause peptic ulcer bleeding [43], which might promote the fecal-oral transmission of HFMD. Our results suggest that children should reduce their outdoor exposure on days with high NO_2_ concentrations. The overall association between the SO_2_ concentration and HFMD cases was nonsignificant, which was inconsistent with positive associations in studies in Ningbo [44], Shenzhen [21], Hefei [45] and Wuhan [16]. Approximately 95% of the SO_2_ concentration data in our study were lower than 30 μg/m^3^, which may lead to a wide confidence interval at high SO_2_ concentrations. Therefore, our results only suggest that the association between low SO_2_ concentrations and HFMD cases was not significant. We are unsure whether the nonsignificant result at high SO_2_ concentrations was due to the wide confidence interval or no actual association between SO_2_ and HFMD. Further studies are needed in areas with high SO_2_ concentrations.

Regarding the heterogeneity of the air pollutant-HFMD associations, we found that demographic compositions and health resources could partly explain the heterogeneity. Among these factors, the proportion of primary school students was an important effect modifier that explained 10.9% and 5.9% of the heterogeneity of the associations of SO_2_ and NO_2_ concentrations with the number of HFMD cases, respectively. A high proportion of primary school students weakened the positive effect of NO_2_ on the number of HFMD cases and made the association between SO_2_ and the number of HFMD cases negative. A possible reason is that the AQI and the concentration of most air pollutants, including SO_2_ and NO_2_, are usually higher in winter. During this time, families with children are more likely to reduce the time spent on outdoor activities because of cold weather and early warning measures [37]. Therefore, people in regions with large proportions of primary school students may take more protective measures, which may reduce exposure to HFMD among children. However, the number of licensed physicians, the number of hospital beds, the population density and the urbanization rate were significant effect modifiers. Their effect modifications on the associations between pollutants and the number of HFMD cases were slight, and their *CIs* on the 10th and 90th percentiles of the modifiers were heavily overlapping. Therefore, it is difficult to say whether these sight modification effects have epidemiological implications. However, these results could still provide clues for future research to explore the modification effects on regions with larger differences in these factors. The results also suggest that children in regions with lower health resources particularly need to reduce their exposure to air pollutants.

Our study explored the heterogeneity of the relationships between air pollutant concentrations and the number of HFMD cases and identified significant effect modifiers. We mainly focused on the modification effects of socioeconomic factors, so we selected cities with different levels of economic development and similar climates to avoid interference from meteorological factors. Therefore, a limitation is that meteorological factors may have modification effects, which were not explored in our study. Additional studies in more regions with diverse climates are needed. For the exposure assignment of each city, we chose the nearest neighbor station to obtain the meteorological exposure data and calculated the arithmetic mean of the monitoring stations to obtain the air pollution exposure data. Therefore, another limitation is that misclassification of exposure may occur in some populations. Further research can consider other better approaches, such as land use regression and random forests, to obtain more accurate exposure data. Finally, when we explored the association between one air pollutant and HFMD cases, other air pollutants were also important confounders. However, some air pollutant data were correlated. Although we controlled for collinearity by not including highly correlated variables in the same model, the moderately correlated variables still influenced the results.

## 5. Conclusions

Our study found that the air pollutant-HFMD associations between cities showed heterogeneity. Different demographic characteristics and health care resources could partially explain the heterogeneity. Susceptible populations in regions with a smaller proportion of primary school students and fewer medical resources should avoid exposure to SO_2_ and NO_2_. Children in regions with lower health resources particularly need to reduce their exposure to air pollutants. The results could be helpful for relevant public health decision-making to prevent HFMD.

## Supporting information

S1 Table Descriptions of daily HFMD cases, meteorological factors and air pollutants in 17 cities. (DOCX)

S2 Table All results of the tests of heterogeneity and effect modifiers of the air pollutant-HFMD relationship. (DOCX)

S3 Table Descriptions of socioeconomic factors for 17 cities from 2015 to 2017. (DOCX)

S1 Fig Locations of 13 meteorological monitoring stations and 86 air pollutant monitoring stations in the basin area of Sichuan Province. (DOCX)

S2 Fig The correlations between air pollutants and meteorological variables. (DOCX)

S3 Fig The overall and city-specific cumulative-response curves of PM2.5-HFMD relationships and predicted PM2.5-HFMD association considering effect modifiers at the 10th and 90th percentiles. (DOCX)

S4 Fig The overall and city-specific cumulative-response curves of AQI-HFMD relationships. (DOCX)

S1 Text The results of sensitivity analysis. Table A. The results of overdispersion test for the HFMD series. Table B. Different confounder model settings. Table C. Different autoregressive term settings. Table D. The values of different knots of splines for the exposure -response structure of air pollutants. Table E. The QAICs of different knots of splines for the exposure -response structure of air pollutants. Fig A. The overall model fit of different values of the degrees of freedom of time splines. Fig B. The overall model fit of different model settings of temperature. Fig C. The overall model fit of different model settings of Humid. Fig D. The overall model fit of different model settings of sunshine hours. Fig E. The overall model fit of different model settings of wind velocity. Fig F. The overall model fit of different model settings of precipitation. Fig G. The autocorrelation analysis on the residuals of the HFMD cases. Fig H. The overall model fit of different autoregressive term settings. Fig I. The ACF and PACF analysis on the residuals of the HFMD cases after controlling autoregressive term. Fig J. The QAICs of different dfs of splines for lag-response structure of air pollutants.
(DOCX)
