# Supplementary material for: Modification effects of socioeconomic factors on associations between air pollutants and hand, foot, and mouth disease: A multicity time-series study based on heavily polluted areas in the basin area of Sichuan Province, China
Source: PLoS Negl Trop Dis. 2022 Nov 22;16(11):e0010896. doi: 10.1371/journal.pntd.0010896 (PMC9681081; doi:10.1371/journal.pntd.0010896)
Supplement: S1 Table — (DOCX) [file pntd.0010896.s001.docx]

S1 Table. Descriptions of daily HFMD cases, meteorological factors and air pollutants in 17 cities from 2015 to 2017

| City | Cases | PM_10_ | PM_2.5_ | SO_2_ | NO_2_ | O_3_ | CO | tm | humid | win | sun | rain |
| --- | --- | --- | --- | --- | --- | --- | --- | --- | --- | --- | --- | --- |
| Chengdu | 86128 | 98.0±61.6 | 58.9±40.0 | 13.3±5.4 | 50.2±15.7 | 66.4±44.6 | 1.0±0.4 | 16.7±7.2 | 81.5± 8.4 | 1.3±0.5 | 3.0± 3.5 | 2.6± 8.5 |
| Zigong | 3603 | 98.7±54.7 | 71.3±44.9 | 16.2±6.0 | 32.2±10.4 | 58.1±27.0 | 0.9±0.3 | 19.2±6.9 | 77.6±11.1 | 1.1±0.4 | 3.0± 3.8 | 2.9± 8.5 |
| Luzhou | 4924 | 85.3±49.4 | 58.9±36.4 | 18.1±9.8 | 30.4±10.8 | 54.5±31.6 | 0.6±0.2 | 18.4±7.0 | 86.6±10.1 | 1.8±0.5 | 3.4± 4.1 | 3.4± 9.4 |
| Deyang | 10659 | 88.0±53.1 | 52.4±35.6 | 11.4±5.1 | 28.7±12.9 | 70.1±39.0 | 0.9±0.3 | 17.9±7.4 | 71.6±12.7 | 1.8±0.6 | 3.5± 3.7 | 2.1±10.7 |
| Mianyang | 11280 | 74.7±47.2 | 47.8±32.4 | 10.6±4.0 | 33.5±11.4 | 59.6±33.9 | 0.9±0.3 | 17.9±7.4 | 71.6±12.7 | 1.8±0.6 | 3.5± 3.7 | 2.1±10.7 |
| Guangyuan | 4273 | 61.1±43.0 | 23.5±19.5 | 19.6±7.8 | 33.0±12.8 | 61.7±34.7 | 0.8±0.4 | 16.5±7.6 | 70.3±14.1 | 1.7±0.7 | 3.8± 3.8 | 2.5± 8.0 |
| Suining | 7891 | 74.9±41.6 | 44.1±27.2 | 12.0±5.4 | 24.1±10.0 | 69.7±38.1 | 0.8±0.3 | 18.1±7.5 | 80.6±12.2 | 1.4±0.5 | 3.2± 3.8 | 2.6± 7.9 |
| Neijiang | 5557 | 77.0±46.2 | 53.9±37.2 | 18.5±9.5 | 27.4±10.1 | 69.8±37.2 | 0.7±0.3 | 18.2±7.3 | 81.3± 9.7 | 1.4±0.5 | 3.2± 3.9 | 2.5± 8.8 |
| Leshan | 6569 | 78.3±48.5 | 54.0±36.5 | 16.0±9.4 | 32.3±10.6 | 62.6±33.9 | 1.0±0.3 | 18.7±6.9 | 74.9±10.7 | 1.2±0.4 | 2.9± 3.7 | 2.8± 7.8 |
| Nanchong | 9789 | 80.7±42.4 | 53.3±29.6 | 11.6±4.7 | 29.7±10.7 | 56.1±27.8 | 0.8±0.3 | 18.1±7.6 | 76.6±13.0 | 1.7±0.8 | 3.7± 4.1 | 2.9±11.7 |
| Meishan | 13338 | 87.0±48.1 | 56.8±36.3 | 13.5±7.4 | 32.1±12.7 | 68.2±41.5 | 0.7±0.3 | 18.7±6.9 | 74.9±10.7 | 1.2±0.4 | 2.9± 3.7 | 2.8± 7.8 |
| Yibing | 4518 | 80.6±47.3 | 57.0±38.5 | 19.6±8.4 | 30.2± 9.9 | 55.2±30.2 | 1.0±0.3 | 19.2±6.9 | 77.6±11.1 | 1.1±0.4 | 3.0± 3.8 | 2.9± 8.5 |
| Guangan | 5497 | 76.9±48.2 | 42.7±32.0 | 17.5±9.0 | 22.9± 8.8 | 67.4±39.5 | 0.9±0.3 | 18.2±7.5 | 79.3±12.6 | 2.1±0.7 | 3.4±10.1 | 3.7± 4.4 |
| Dazhou | 9283 | 83.7±50.8 | 56.0±38.9 | 11.8±4.9 | 39.3± 9.5 | 51.6±24.5 | 1.0±0.5 | 18.4±7.8 | 76.9±11.1 | 1.2±0.3 | 3.4± 4.0 | 3.4±10.3 |
| Yaan | 3830 | 72.4±44.5 | 42.9±28.2 | 13.0±5.4 | 25.5±7.0 | 60.1±24.0 | 0.9±0.4 | 17.2±6.9 | 79.0±10.6 | 0.9±0.4 | 2.4± 3.3 | 4.1±11.1 |
| Bazhong | 7625 | 56.7±37.6 | 34.9±26.3 | 5.0±2.7 | 25.9±8.6 | 52.1±31.9 | 0.9±0.3 | 17.8±7.7 | 74.4±10.9 | 1.3±0.5 | 4.5± 4.5 | 2.8± 8.8 |
| Ziyang | 6271 | 83.5±46.1 | 40.1±29.4 | 18.6±14.4 | 20.8±8.2 | 76.5±36.1 | 0.7±0.3 | 18.2±7.3 | 81.3± 9.7 | 1.4±0.5 | 3.2± 3.9 | 2.5± 8.8 |
