# Supplementary material for: Modification effects of socioeconomic factors on associations between air pollutants and hand, foot, and mouth disease: A multicity time-series study based on heavily polluted areas in the basin area of Sichuan Province, China
Source: PLoS Negl Trop Dis. 2022 Nov 22;16(11):e0010896. doi: 10.1371/journal.pntd.0010896 (PMC9681081; doi:10.1371/journal.pntd.0010896)
Supplement: S2 Table — (DOCX) [file pntd.0010896.s002.docx]

S2 Table. Heterogeneity and all potential effect modifiers of the relationships between pollutant concentrations and the number of HFMD cases

| Meta-predictor | LR test | | | Model fit | Cochran Q test | | | *I^2^* |
| --- | --- | --- | --- | --- | --- | --- | --- | --- |
|  | *χ^2^* statistic | *df* | *P* | AIC | *χ^2^* statistic | *df* | *P* | (%) |
| PM_10_ |  |  |  |  |  |  |  |  |
| Intercept only | - | - | - | 170.1 | 115.5 | 48 | <0.001 | 58.5 |
| GDP per person | 6.8 | 3 | 0.079 | 169.4 | 100.5 | 45 | <0.001 | 55.2 |
| GDP increase | 4.5 | 3 | 0.212 | 171.6 | 104.4 | 45 | <0.001 | 56.9 |
| Urbanization rate | 6.9 | 3 | 0.075 | 169.2 | 99.9 | 45 | <0.001 | 55.0 |
| Population density | 1.5 | 3 | 0.682 | 174.7 | 105.9 | 45 | <0.001 | 57.5 |
| Birth rate | 5.1 | 3 | 0.165 | 171.1 | 104.2 | 45 | <0.001 | 56.8 |
| Proportion of students | 5.6 | 3 | 0.133 | 170.5 | 108.8 | 45 | <0.001 | 58.7 |
| Hospital beds | 2.7 | 3 | 0.440 | 173.4 | 101.2 | 45 | <0.001 | 55.5 |
| Licensed physicians | 8.2 | 3 | 0.042 | 168.0 | 98.4 | 45 | <0.001 | 54.3 |
| Passengers | 3.7 | 3 | 0.296 | 172.4 | 103.9 | 45 | <0.001 | 56.7 |
| SO_2_ |  |  |  |  |  |  |  |  |
| Intercept only | - | - | - | 150.0 | 93.0 | 48 | <0.001 | 48.4 |
| GDP per person | 0.4 | 3 | 0.94 | 155.7 | 91.3 | 45 | <0.001 | 50.7 |
| GDP increase | 5.2 | 3 | 0.158 | 150.9 | 86.4 | 45 | <0.001 | 47.9 |
| Urbanization rate | 0.7 | 3 | 0.873 | 155.4 | 90.4 | 45 | <0.001 | 50.2 |
| Population density | 3.2 | 3 | 0.362 | 152.9 | 86.1 | 45 | <0.001 | 47.7 |
| Birth rate | 0.3 | 3 | 0.960 | 155.8 | 92.4 | 45 | <0.001 | 51.3 |
| Proportion of students | 16.5 | 3 | 0.001 | 139.6 | 72.1 | 45 | 0.006 | 37.5 |
| Hospital beds | 2.9 | 3 | 0.407 | 153.2 | 91.7 | 45 | <0.001 | 50.9 |
| Licensed physicians | 0.6 | 3 | 0.896 | 155.5 | 91.8 | 45 | <0.001 | 51.0 |
| Passengers | 0.3 | 3 | 0.960 | 155.8 | 91.3 | 45 | <0.001 | 50.7 |
| NO_2_ |  |  |  |  |  |  |  |  |
| Intercept only | - | - | - | 154.5 | 98.0 | 48 | <0.001 | 51.0 |
| GDP per person | 0.7 | 3 | 0.873 | 159.7 | 97.0 | 45 | <0.001 | 53.6 |
| GDP increase | 2.4 | 3 | 0.494 | 158.0 | 94.7 | 45 | <0.001 | 52.5 |
| Urbanization rate | 1.1 | 3 | 0.777 | 159.3 | 96.8 | 45 | <0.001 | 53.5 |
| Population density | 3.5 | 3 | 0.321 | 157.0 | 91.9 | 45 | <0.001 | 51.1 |
| Birth rate | 3.3 | 3 | 0.348 | 157.1 | 92.7 | 45 | <0.001 | 51.4 |
| Proportion of students | 11.6 | 3 | 0.009 | 148.9 | 82.0 | 45 | 0.001 | 45.1 |
| Hospital beds | 4.7 | 3 | 0.195 | 155.8 | 94.5 | 45 | <0.001 | 52.4 |
| Licensed physicians | 1.1 | 3 | 0.777 | 159.4 | 96.4 | 45 | <0.001 | 53.3 |
| Passengers | 0.9 | 3 | 0.825 | 159.5 | 97.0 | 45 | <0.001 | 53.6 |
| O_3_ |  |  |  |  |  |  |  |  |
| Intercept only | - | - | - | 131.4 | 108.2 | 48 | <0.001 | 55.6 |
| GDP per person | 5.6 | 3 | 0.133 | 131.7 | 90.0 | 45 | <0.001 | 50.0 |
| GDP increase | 3.6 | 3 | 0.308 | 133.8 | 95.4 | 45 | <0.001 | 52.8 |
| Urbanization rate | 8.7 | 3 | 0.034 | 128.7 | 85.6 | 45 | <0.001 | 47.4 |
| Population density | 7.8 | 3 | 0.050 | 129.6 | 86.9 | 45 | <0.001 | 48.2 |
| Birth rate | 3.6 | 3 | 0.308 | 133.8 | 93.5 | 45 | <0.001 | 51.9 |
| Proportion of students | 7.7 | 3 | 0.053 | 129.6 | 92.6 | 45 | <0.001 | 51.4 |
| Hospital beds | 10.2 | 3 | 0.017 | 127.2 | 98.0 | 45 | <0.001 | 54.1 |
| Licensed physicians | 6.4 | 3 | 0.094 | 130.9 | 90.3 | 45 | <0.001 | 50.2 |
| Passengers | 7.3 | 3 | 0.063 | 130.0 | 85.9 | 45 | <0.001 | 47.6 |
| CO |  |  |  |  |  |  |  |  |
| Intercept only | - | - | - | 154.3 | 102.1 | 48 | <0.001 | 53.0 |
| GDP per person | 2.6 | 3 | 0.457 | 157.7 | 93.1 | 45 | <0.001 | 51.7 |
| GDP increase | 5.2 | 3 | 0.158 | 155.1 | 91.7 | 45 | <0.001 | 50.9 |
| Urbanization rate | 4.0 | 3 | 0.261 | 156.3 | 90.3 | 45 | <0.001 | 50.2 |
| Population density | 4.8 | 3 | 0.187 | 155.5 | 92.1 | 45 | <0.001 | 51.1 |
| Birth rate | 5.8 | 3 | 0.122 | 154.5 | 88.4 | 45 | <0.001 | 49.1 |
| Proportion of students | 3.7 | 3 | 0.296 | 156.6 | 97.5 | 45 | <0.001 | 53.9 |
| Hospital beds | 0.9 | 3 | 0.825 | 159.4 | 98.1 | 45 | <0.001 | 54.1 |
| Licensed physicians | 3.3 | 3 | 0.348 | 157.0 | 91.5 | 45 | <0.001 | 50.8 |
| Passengers | 4.9 | 3 | 0.179 | 155.4 | 90.4 | 45 | <0.001 | 50.2 |
| PM_2.5_ |  |  |  |  |  |  |  |  |
| Intercept only | - | - | - | 194.9 | 131.8 | 64 | <0.001 | 51.4 |
| GDP per person | 1.8 | 4 | 0.772 | 201.1 | 126.8 | 60 | <0.001 | 52.7 |
| GDP increase | 10.6 | 4 | 0.031 | 192.3 | 117.8 | 60 | <0.001 | 49.1 |
| Urbanization rate | 3.0 | 4 | 0.558 | 199.9 | 124.8 | 60 | <0.001 | 51.9 |
| Population density | 0.5 | 4 | 0.974 | 202.4 | 128.6 | 60 | <0.001 | 53.3 |
| Birth rate | 1.9 | 4 | 0.754 | 201.0 | 127.0 | 60 | <0.001 | 52.8 |
| Proportion of students | 5.3 | 4 | 0.258 | 197.6 | 125.4 | 60 | <0.001 | 52.1 |
| Hospital beds | 5.0 | 4 | 0.287 | 197.9 | 122.8 | 60 | <0.001 | 51.2 |
| Licensed physicians | 2.9 | 4 | 0.575 | 200.0 | 125.3 | 60 | <0.001 | 52.1 |
| Passengers | 1.5 | 4 | 0.827 | 201.4 | 125.4 | 60 | <0.001 | 52.1 |
| AQI |  |  |  |  |  |  |  |  |
| Intercept only | - | - | - | 117.0 | 99.8 | 48 | <0.001 | 51.9 |
| GDP per person | 4.9 | 3 | 0.179 | 118.0 | 83.9 | 45 | <0.001 | 46.4 |
| GDP increase | 3.7 | 3 | 0.296 | 119.3 | 88.0 | 45 | <0.001 | 48.9 |
| Urbanization rate | 6.6 | 3 | 0.086 | 116.4 | 81.6 | 45 | 0.001 | 44.8 |
| Population density | 5.6 | 3 | 0.133 | 117.3 | 84.5 | 45 | <0.001 | 46.8 |
| Birth rate | 6.4 | 3 | 0.094 | 116.6 | 85.7 | 45 | <0.001 | 47.5 |
| Proportion of students | 5.5 | 3 | 0.139 | 117.5 | 93.0 | 45 | <0.001 | 51.6 |
| Hospital beds | 3.7 | 3 | 0.296 | 119.2 | 88.9 | 45 | <0.001 | 49.4 |
| Licensed physicians | 5.6 | 3 | 0.133 | 117.3 | 82.9 | 45 | <0.001 | 45.7 |
| Passengers | 4.5 | 3 | 0.212 | 118.5 | 84.4 | 45 | <0.001 | 46.7 |

*“-”means the intercept was not tested by the LR test.
