# Supplementary material for: Modification effects of socioeconomic factors on associations between air pollutants and hand, foot, and mouth disease: A multicity time-series study based on heavily polluted areas in the basin area of Sichuan Province, China
Source: PLoS Negl Trop Dis. 2022 Nov 22;16(11):e0010896. doi: 10.1371/journal.pntd.0010896 (PMC9681081; doi:10.1371/journal.pntd.0010896)
Supplement: S3 Table — (DOCX) [file pntd.0010896.s003.docx]

S3 Table. Descriptions of socioeconomic factors for 17 cities from 2015 to 2017.

| city | GDP per person (CNY) | GDP increase (%) | Birth rate (‰) | Urbanization rate (%) | Population density (people per km2) | Proportion of students (‰) | Passengers (trips) | Hospital beds(per 1000 population) | Licensed physicians (per 1000 population) |
| --- | --- | --- | --- | --- | --- | --- | --- | --- | --- |
| Chengdu | 79381.33 | 28.59165 | 11.30 | 71.31 | 1146.15 | 53.30 | 1167754.00 | 2.90 | 12.1 |
| Zigong | 44036.67 | 14.78073 | 10.16 | 49.31 | 643.11 | 63.28 | 165834.30 | 2.63 | 8.17 |
| Luzhou | 34410.33 | 23.67864 | 10.15 | 47.51 | 351.74 | 92.91 | 535543.70 | 2.63 | 7.47 |
| Deyang | 50381.00 | 17.93987 | 9.72 | 49.68 | 596.15 | 49.61 | 246424.30 | 2.43 | 8.00 |
| Mianyang | 38990.33 | 22.14808 | 9.52 | 49.50 | 237.56 | 54.21 | 293040.70 | 2.77 | 8.03 |
| Guangyuan | 25329.33 | 22.02043 | 10.30 | 42.40 | 162.08 | 56.40 | 162769.30 | 3.17 | 8.4 |
| Suining | 31106.00 | 20.92562 | 8.62 | 47.15 | 615.36 | 50.69 | 176439.00 | 2.47 | 6.57 |
| Neijiang | 34089.33 | 24.26813 | 9.92 | 46.74 | 695.82 | 61.39 | 424463.00 | 2.33 | 6.47 |
| Leshan | 43071.00 | 11.13901 | 9.82 | 48.74 | 256.76 | 54.10 | 185247.00 | 2.57 | 7.57 |
| Nanchong | 26089.33 | 15.87421 | 8.78 | 45.12 | 512.35 | 56.60 | 430941.70 | 2.57 | 7.07 |
| Meishan | 37070.33 | 20.55995 | 11.22 | 43.34 | 419.12 | 49.46 | 157713.30 | 2.27 | 7.07 |
| Yibing | 37221.00 | 14.90397 | 10.55 | 46.62 | 340.15 | 83.92 | 272390.30 | 2.57 | 7.53 |
| Guangan | 33403.33 | 21.05839 | 9.15 | 38.76 | 513.33 | 75.55 | 144909.30 | 2.10 | 6.47 |
| Dazhou | 26110.00 | 16.72418 | 9.95 | 42.4 | 338.92 | 73.71 | 281297.00 | 1.90 | 6.17 |
| Yaan | 35676.67 | 17.26287 | 9.47 | 43.95 | 102.27 | 60.46 | 91154.67 | 3.07 | 8.63 |
| Bazhong | 16543.00 | 19.93513 | 9.83 | 39.05 | 269.92 | 58.11 | 220133.30 | 2.17 | 6.57 |
| Ziyang | 37715.67 | 19.96649 | 9.69 | 40.31 | 445.13 | 75.57 | 234230.70 | 3.40 | 7.37 |
