# Supplementary material for: Modification effects of socioeconomic factors on associations between air pollutants and hand, foot, and mouth disease: A multicity time-series study based on heavily polluted areas in the basin area of Sichuan Province, China
Source: PLoS Negl Trop Dis. 2022 Nov 22;16(11):e0010896. doi: 10.1371/journal.pntd.0010896 (PMC9681081; doi:10.1371/journal.pntd.0010896)
Supplement: S1 Fig — (DOCX) [file pntd.0010896.s004.docx]

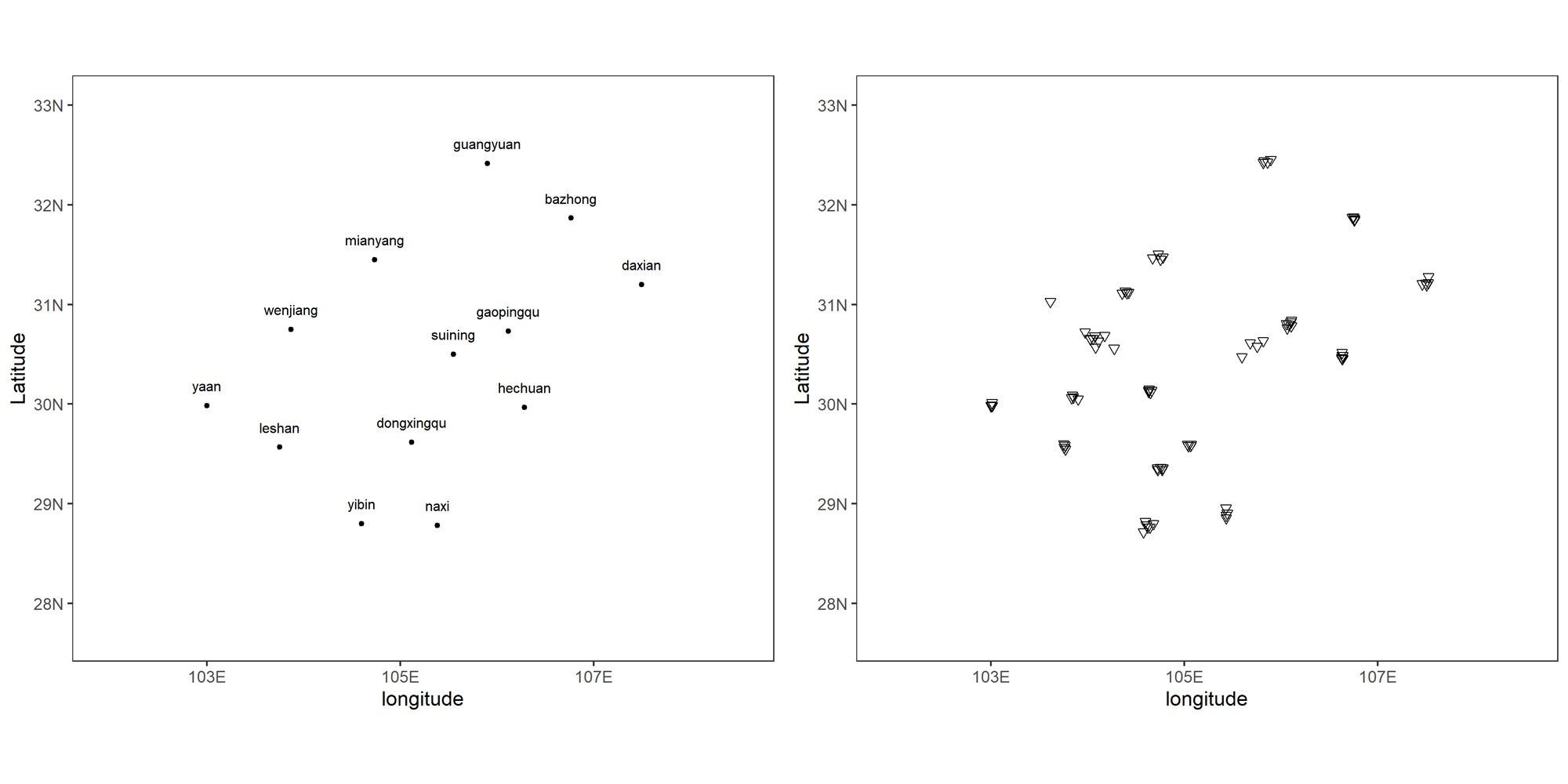


S1 Fig. Locations of 13 meteorological monitoring stations (Left) and 86 air pollutant monitoring stations (Right) in the basin area of Sichuan Province.

For meteorological factors, we chose the neighbor station substitution method, which means the data from the nearest meteorological monitoring station to the geometric center of the city was chosen to represent the exposure of that prefecture-level city. For air pollutant data, the arithmetic mean of the 24 hourly concentration values was taken as the daily average concentration of pollutants for each monitoring station. Then, the arithmetic mean of the daily average concentration of effective monitoring stations within each prefecture-level city was the daily average concentration of pollutants in that city.
