# Supplementary material for: Modification effects of socioeconomic factors on associations between air pollutants and hand, foot, and mouth disease: A multicity time-series study based on heavily polluted areas in the basin area of Sichuan Province, China
Source: PLoS Negl Trop Dis. 2022 Nov 22;16(11):e0010896. doi: 10.1371/journal.pntd.0010896 (PMC9681081; doi:10.1371/journal.pntd.0010896)
Supplement: S2 Fig — (DOCX) [file pntd.0010896.s005.docx]

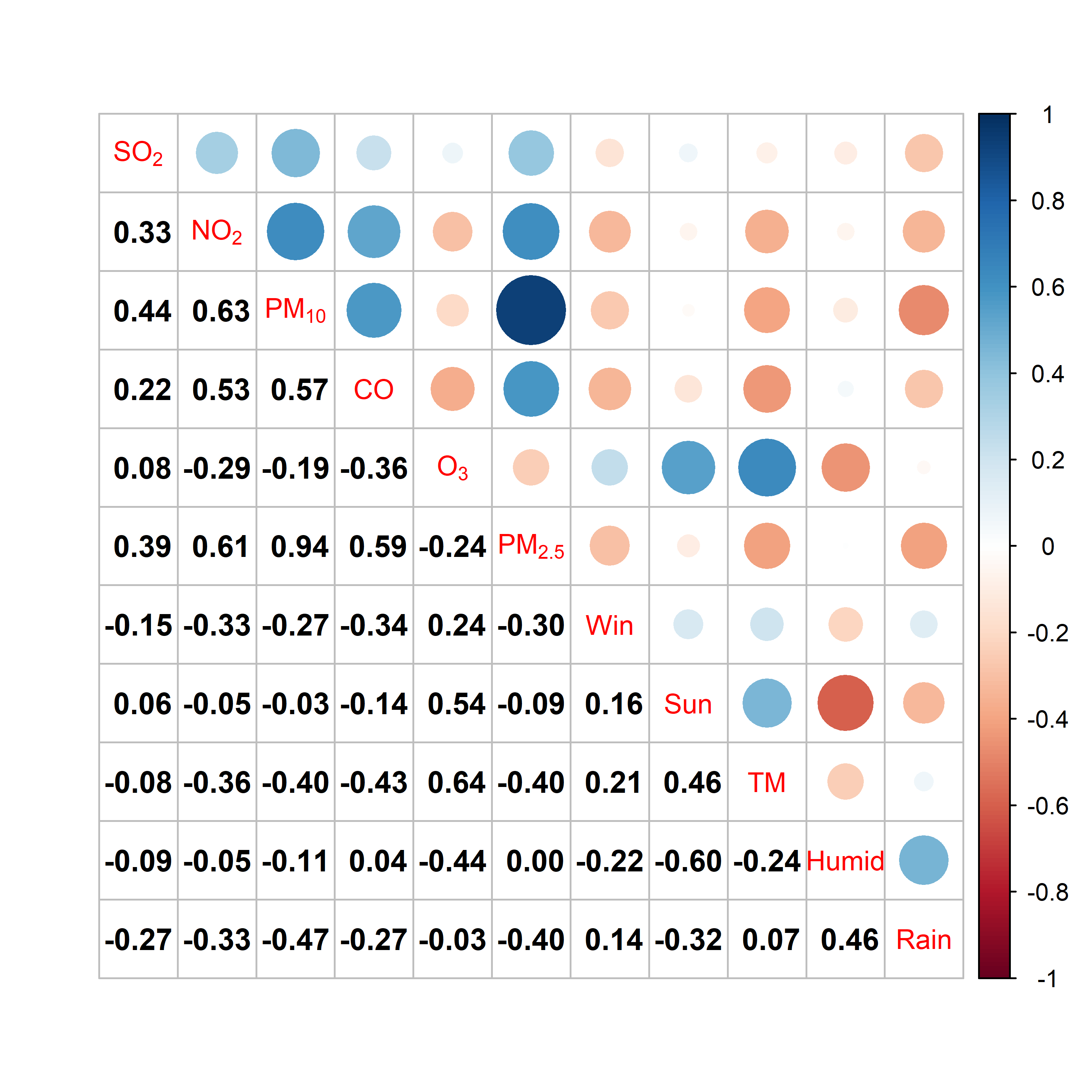
 S2 Fig. The correlation between air pollutants and meteorological variables for

seventeen cities in Sichuan Province from 2015 to 2017.
