# Supplementary material for: Modification effects of socioeconomic factors on associations between air pollutants and hand, foot, and mouth disease: A multicity time-series study based on heavily polluted areas in the basin area of Sichuan Province, China
Source: PLoS Negl Trop Dis. 2022 Nov 22;16(11):e0010896. doi: 10.1371/journal.pntd.0010896 (PMC9681081; doi:10.1371/journal.pntd.0010896)
Supplement: S1 Text — Table A. The results of overdispersion test for the HFMD series. Table B. Different confounder model settings. Table C. Different autoregressive term settings. Table D. The values of different knots of splines for the exposure -response structure of air pollutants. Table E. The QAICs of different knots of splines for the exposure -response structure of air pollutants. Fig A. The overall model fit of different values of the degrees of freedom of time splines. Fig B. The overall model fit of different model settings of temperature. Fig C. The overall model fit of different model settings of Humid. Fig D. The overall model fit of different model settings of sunshine hours. Fig E. The overall model fit of different model settings of wind velocity. Fig F. The overall model fit of different model settings of precipitation. Fig G. The autocorrelation analysis on the residuals of the HFMD cases. Fig H. The overall model fit of different autoregressive term settings. Fig I. The ACF and PACF analysis on the residuals of the HFMD cases after controlling autoregressive term. Fig J. The QAICs of different dfs of splines for lag-response structure of air pollutants. (DOCX) [file pntd.0010896.s008.docx]

**Sensitivity Analysis:**

1. *The choice of model.*

We considered using a Poisson regression model because of the characteristics of the HFMD incidence in the population. However, the application of Poisson regression required the condition of equal dispersion to be met, so we conducted an overdispersion test for the HFMD series of each city. The results (Table A) revealed that the HFMD series were overdispersion, so we finally adopted the quasi-Poisson regression model.

Table A. The results of overdispersion test for the HFMD series

| City | Statistic | *P*-value |
| --- | --- | --- |
| Chengdu | 31656.8 | *P*<0.05 |
| Zigong | 2885.7 | *P*<0.05 |
| Luzhou | 3820.8 | *P*<0.05 |
| Deyang | 6066.0 | *P*<0.05 |
| Mianyang | 5392.2 | *P*<0.05 |
| Guangyuan | 4054.6 | *P*<0.05 |
| Suining | 3400.3 | *P*<0.05 |
| Neijiang | 3389.5 | *P*<0.05 |
| Leshan | 3831.2 | *P*<0.05 |
| Nanchong | 6108.7 | *P*<0.05 |
| Meishan | 7162.2 | *P*<0.05 |
| Yibing | 2392.3 | *P*<0.05 |
| Guangan | 4903.7 | *P*<0.05 |
| Dazhou | 5510.5 | *P*<0.05 |
| Yaan | 4845.9 | *P*<0.05 |
| Bazhong | 6654.7 | *P*<0.05 |
| Ziyang | 3973.0 | *P*<0.05 |

1. *The choice of degrees of freedom (dfs)* *to control for seasonal and long-term trends.*

To control for seasonal and long-term trends, we selected a natural cubic spline and changed the degrees of freedom from 1 to 10 per year. QAICs, the sum of QAIC of 17cities, was used to evaluate the goodness of fit of the model. The result (Fig A) showed the value of QAICs decreased with degrees of freedom. When *df* exceeded 8, the values of QAICs was stable and much smaller than it in 1-7 *df*. So, considering goodness of fit and simplicity of the model, we chose a natural cubic spline with 8*df* per year.


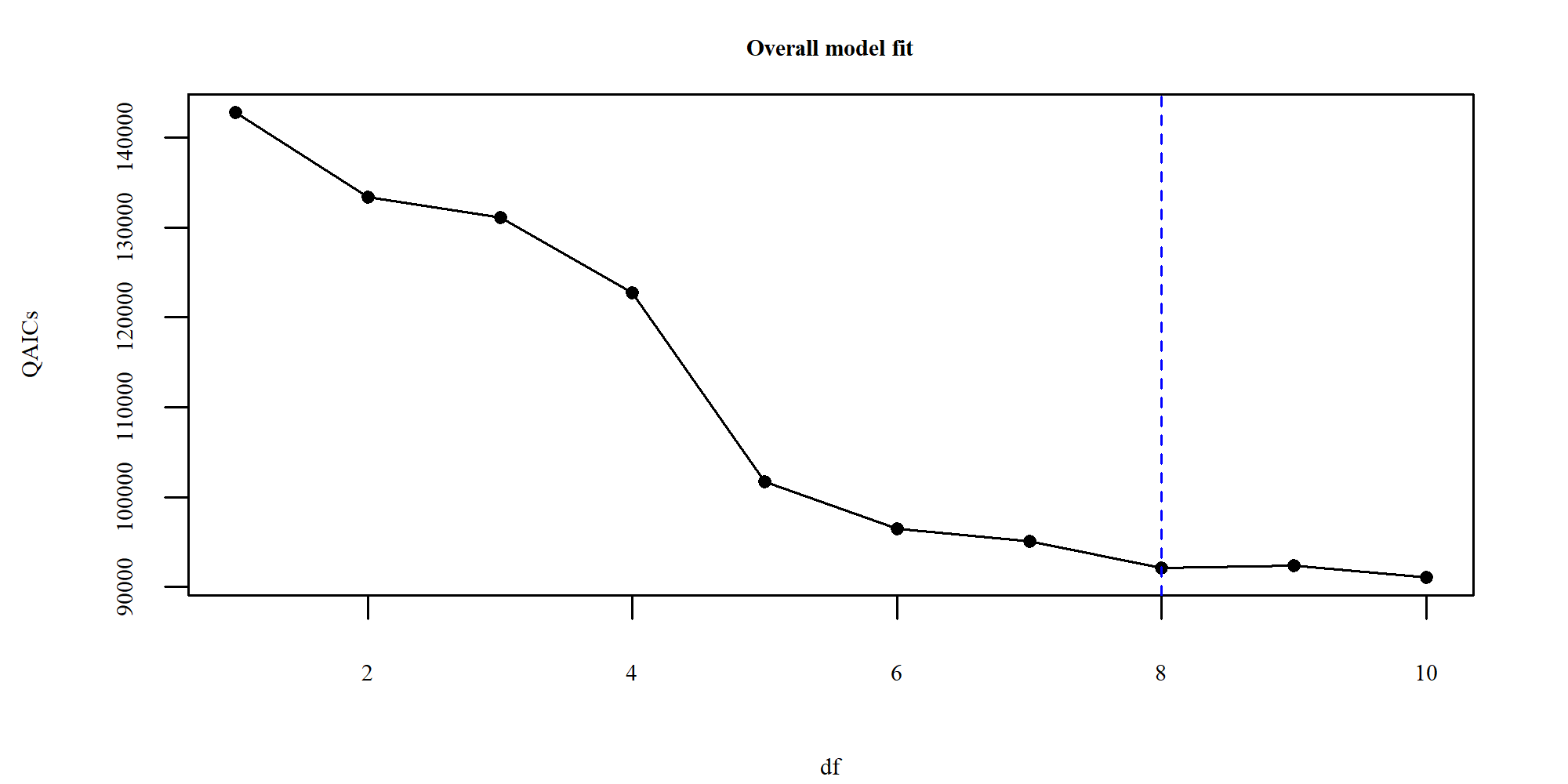


Fig A. The overall model fit of different values of the degrees of freedom of time splines.

1. *The choice of the form of meteorological confounders.*

To control meteorological confounding effects, we included mean temperature, mean relative humidity, mean wind velocity, precipitation and sunshine hours to the model. We set up 9 models for each factor (Table B). C0 means the model with no confounder. Fig B.- Fig F. show the model fitting results for meteorological variables. We selected C6 for temperature and C2 for relative humidity, sunshine hours, wind velocity and precipitation because of smaller values of QAICs. Finally, mean temperature was included by calculating simple moving weighted averages and a natural cubic spline with 3 *df*. Relative humidity, sunshine hours, wind velocity and precipitation were incorporated by calculating simple moving weighted averages.

Table B. Different confounder model settings

| Notation | Exposure-response | Lag days | Lag model |
| --- | --- | --- | --- |
| C0 | - | - | - |
| C1 | Linear relationship | 4 | - |
| C2 | Linear relationship | 4-10 | Simple moving average |
| C3 | Linear relationship | 4-10 | Exponential moving average |
| C4 | Linear relationship | 4-10 | natural cubic splines with 4 *df* |
| C5 | Nonlinear relationship | 4 | - |
| C6 | Nonlinear relationship | 4-10 | Simple moving average |
| C7 | Nonlinear relationship | 4-10 | Exponential moving average |
| C8 | Nonlinear relationship | 4-10 | natural cubic splines with 4 *df* |


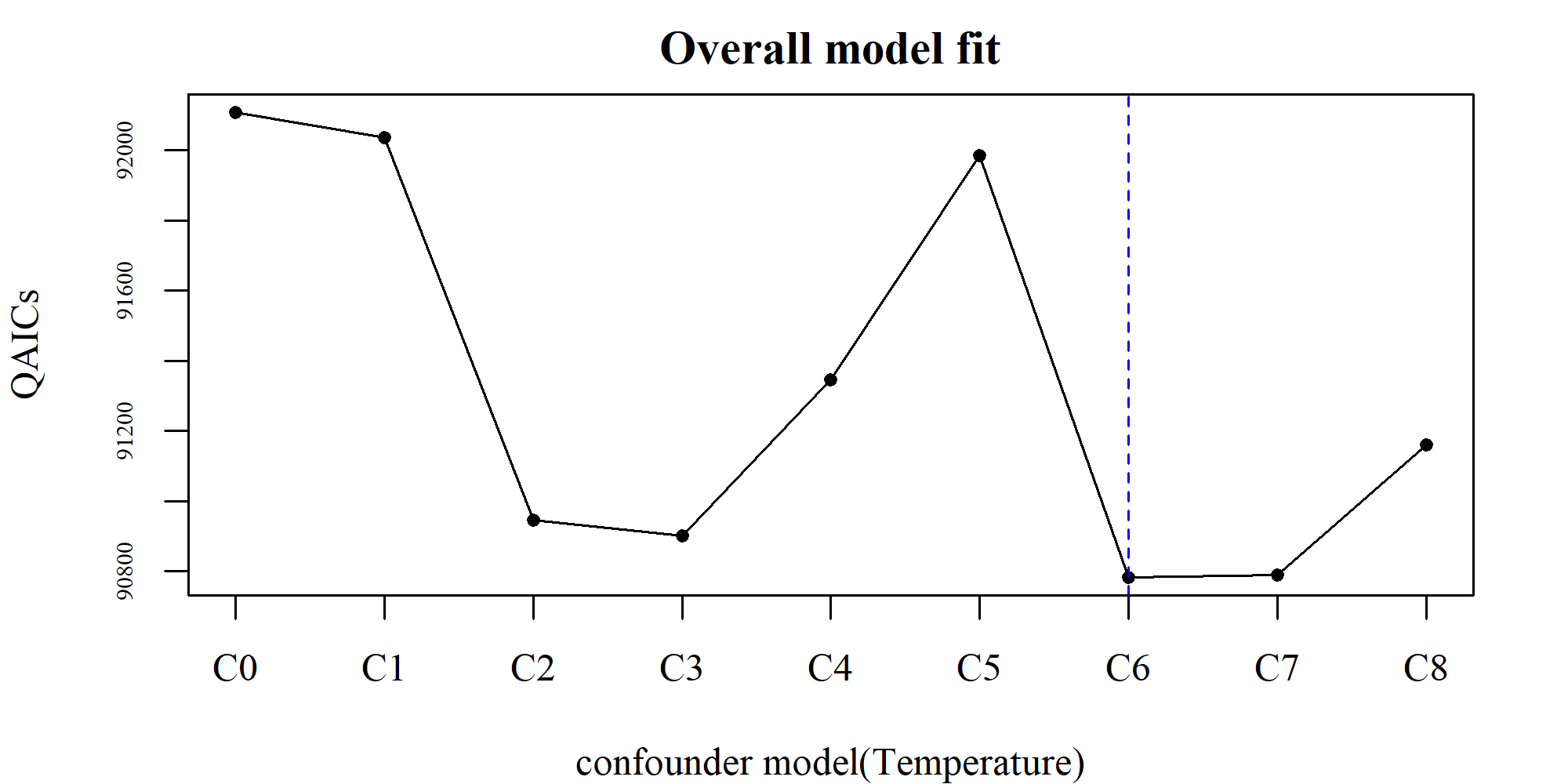


Fig B. The overall model fit of different model settings of temperature.


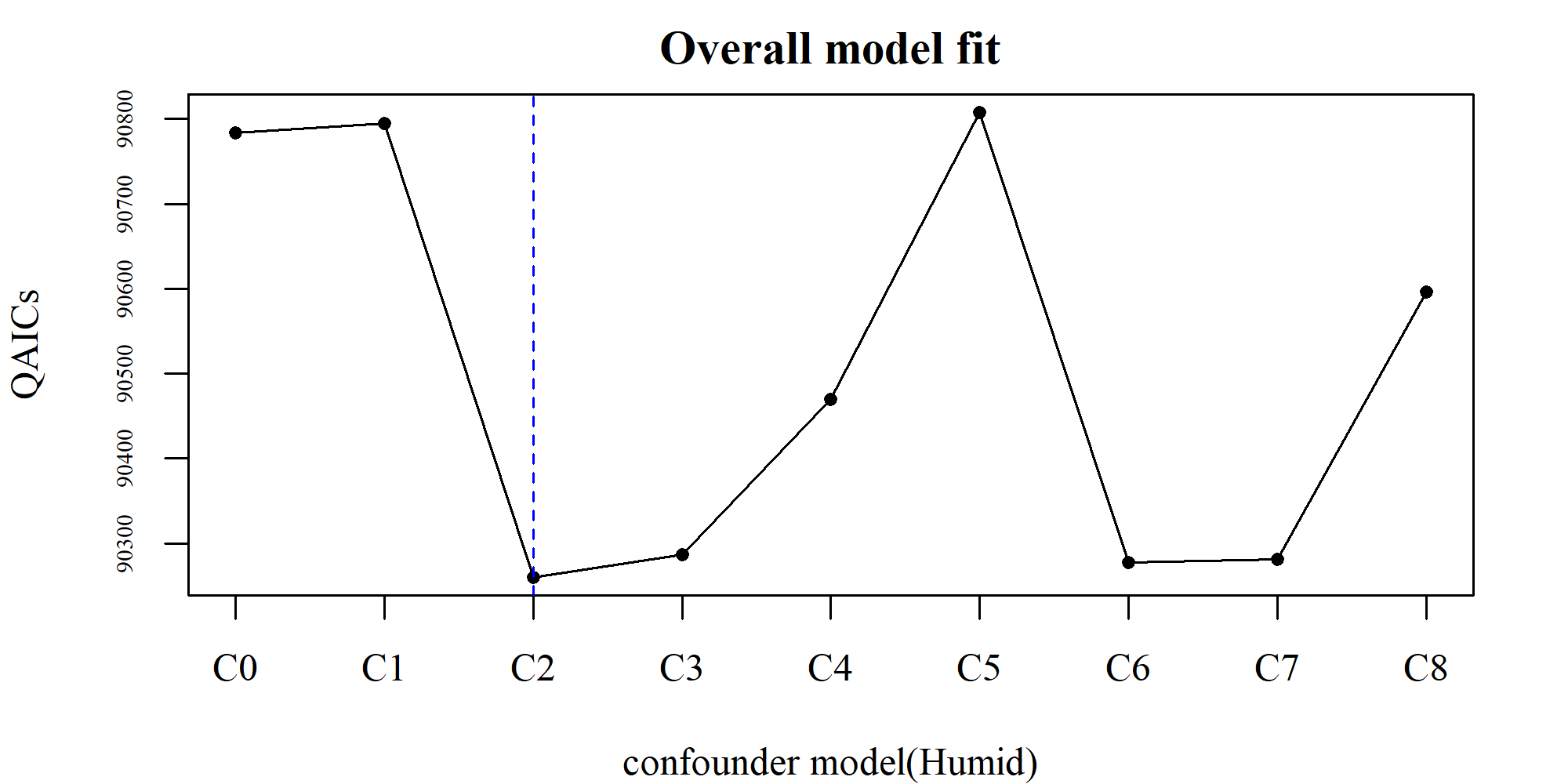


Fig C. The overall model fit of different model settings of Humid.


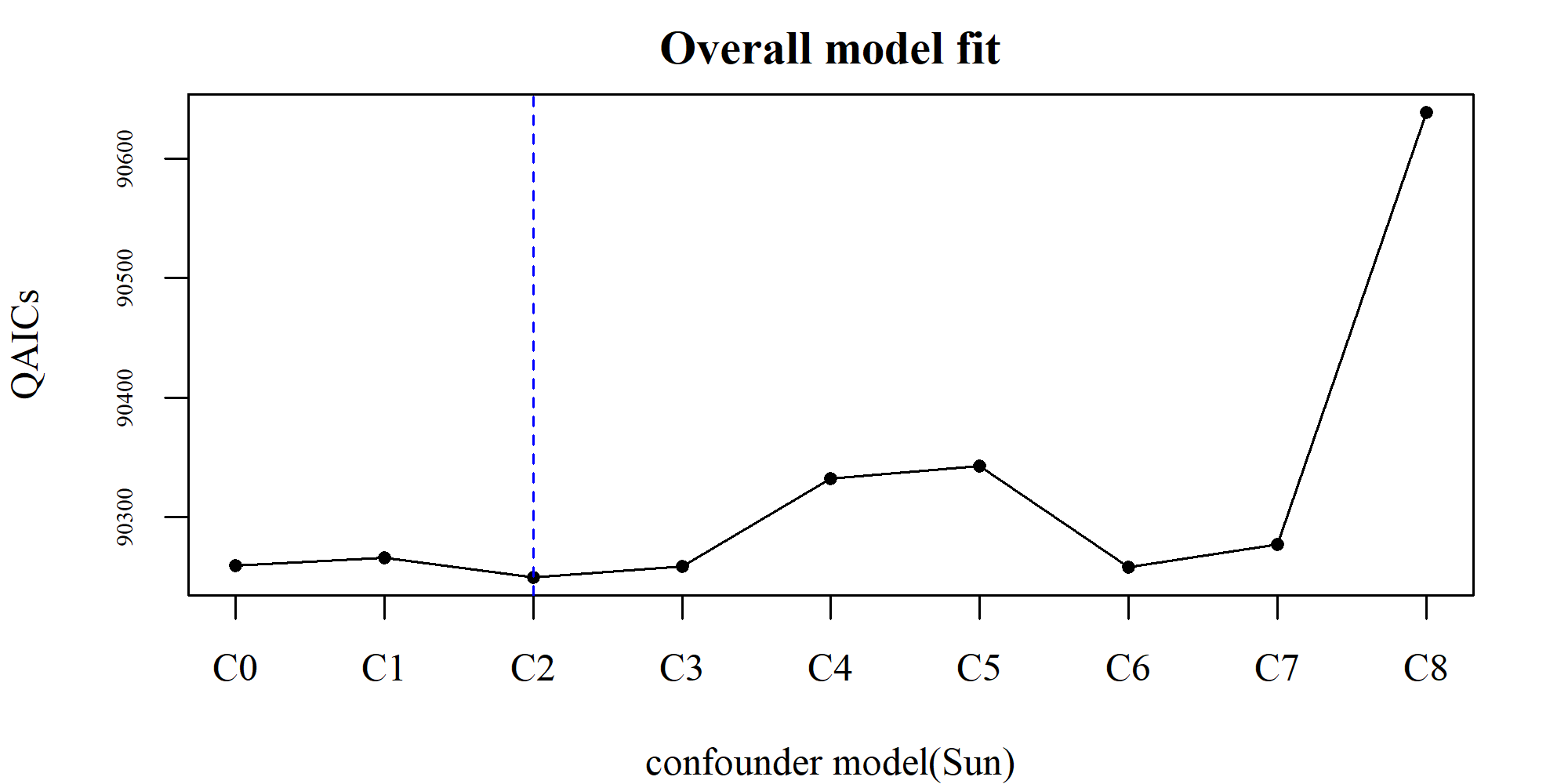


Fig D. The overall model fit of different model settings of sunshine hours.


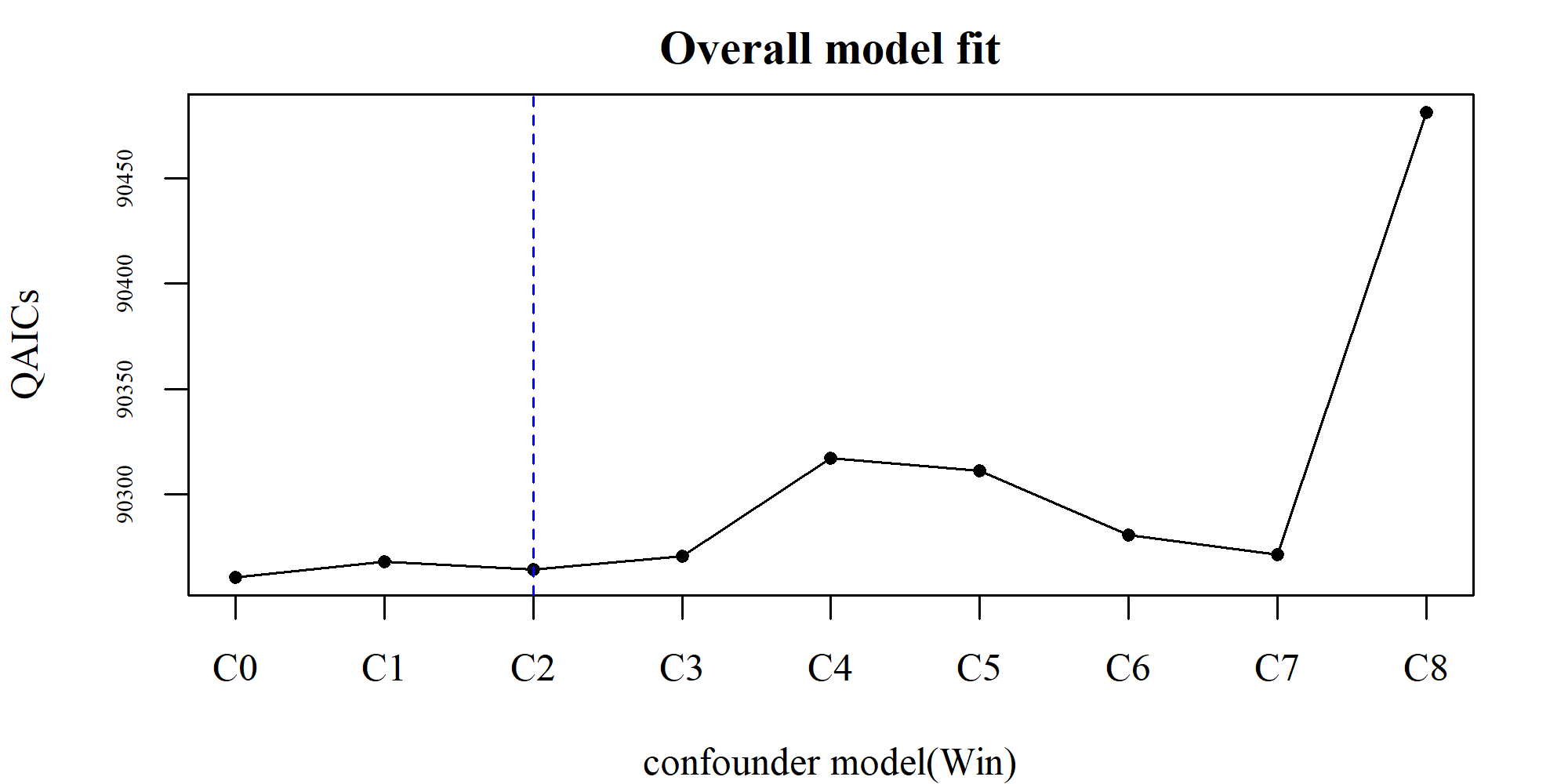


Fig E. The overall model fit of different model settings of wind velocity.

\
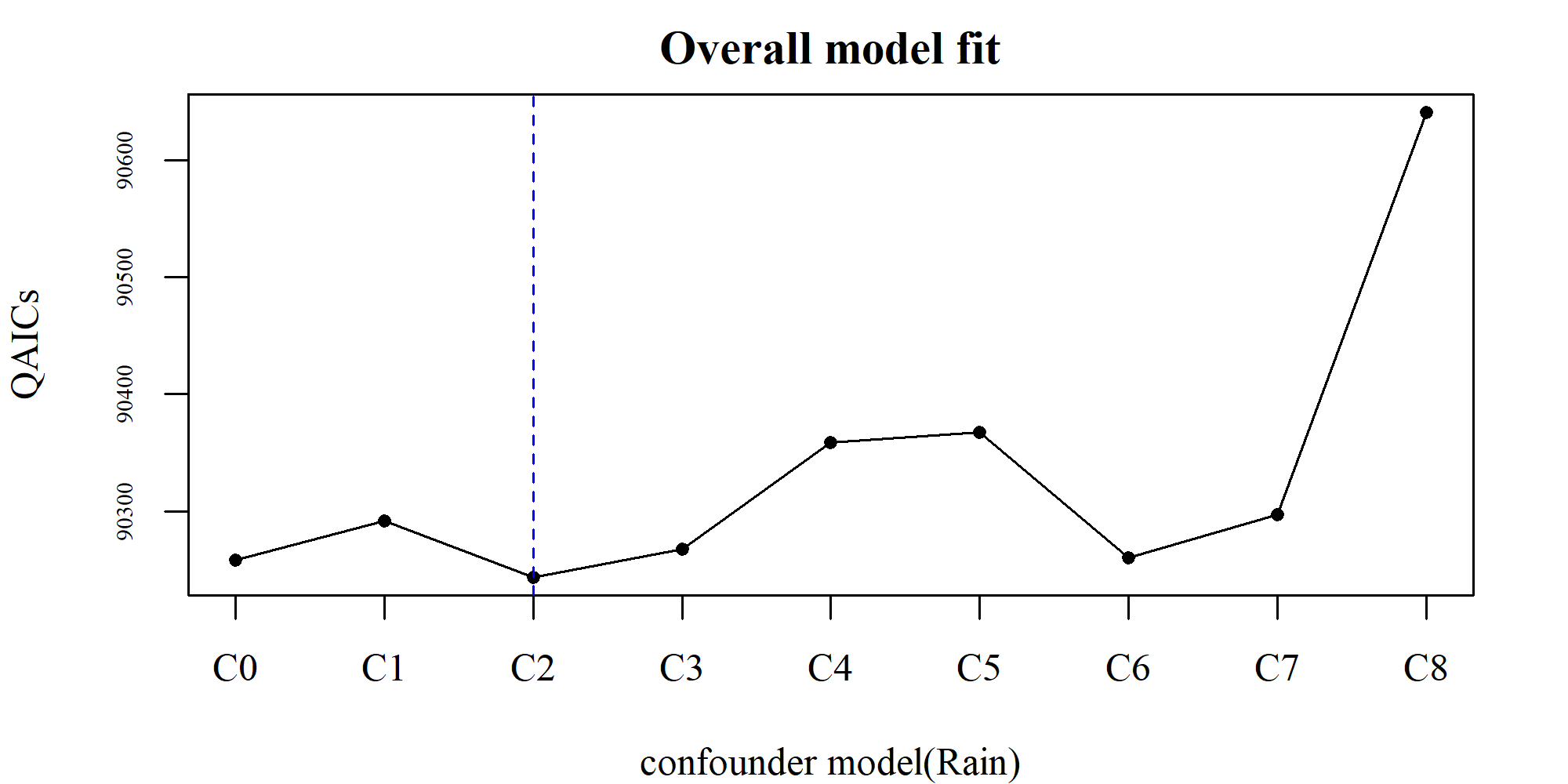


Fig F. The overall model fit of different model settings of precipitation.

1. *The choice of the form of the autoregressive term.*

To explore the autocorrelation of HFMD cases series, we performed an autocorrelation analysis on the residuals of the HFMD time series after controlling seasonal and long-term trends. Fig G showed the residuals series was autocorrelated at lag 1 and lag 2.

Therefore, we set up 6 autoregressive terms to control for autocorrelations of the number of HFMD cases (Table C). Fig H shows the fitting results of different terms. The value of QAICs for A2 was much smaller than others. Therefore, we finally chose the autoregressive term of daily HFMD cases on logarithmic scale with lag 1 and 2 days. After incorporating A2, the autocorrelation of the residuals was much smaller (Fig I).


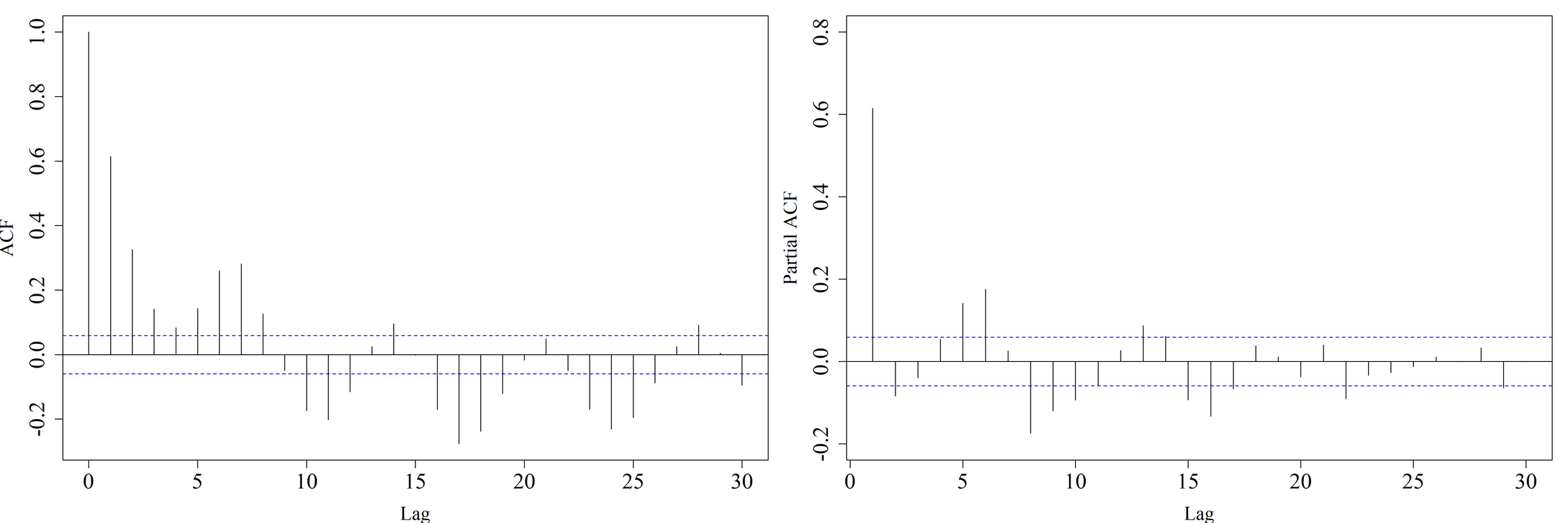


Fig G. The autocorrelation analysis on the residuals of the HFMD cases

Table C. Different autoregressive term settings

| Notation | Lag steps | Scale | Limited settings |
| --- | --- | --- | --- |
| A0 | - | - | - |
| A1 | Lag 1~2 | Original scale | None |
| A2 | Lag 1~2 | Logarithmic scale | None |
| A3 | Lag 1~2 | Logarithmic scale | Simple moving average |
| A4 | Lag 4~10 | Original scale | None |
| A5 | Lag 4~10 | Logarithmic scale | natural cubic splines with 4 *df* |
| A6 | Lag 4~10 | Logarithmic scale | Simple moving average |


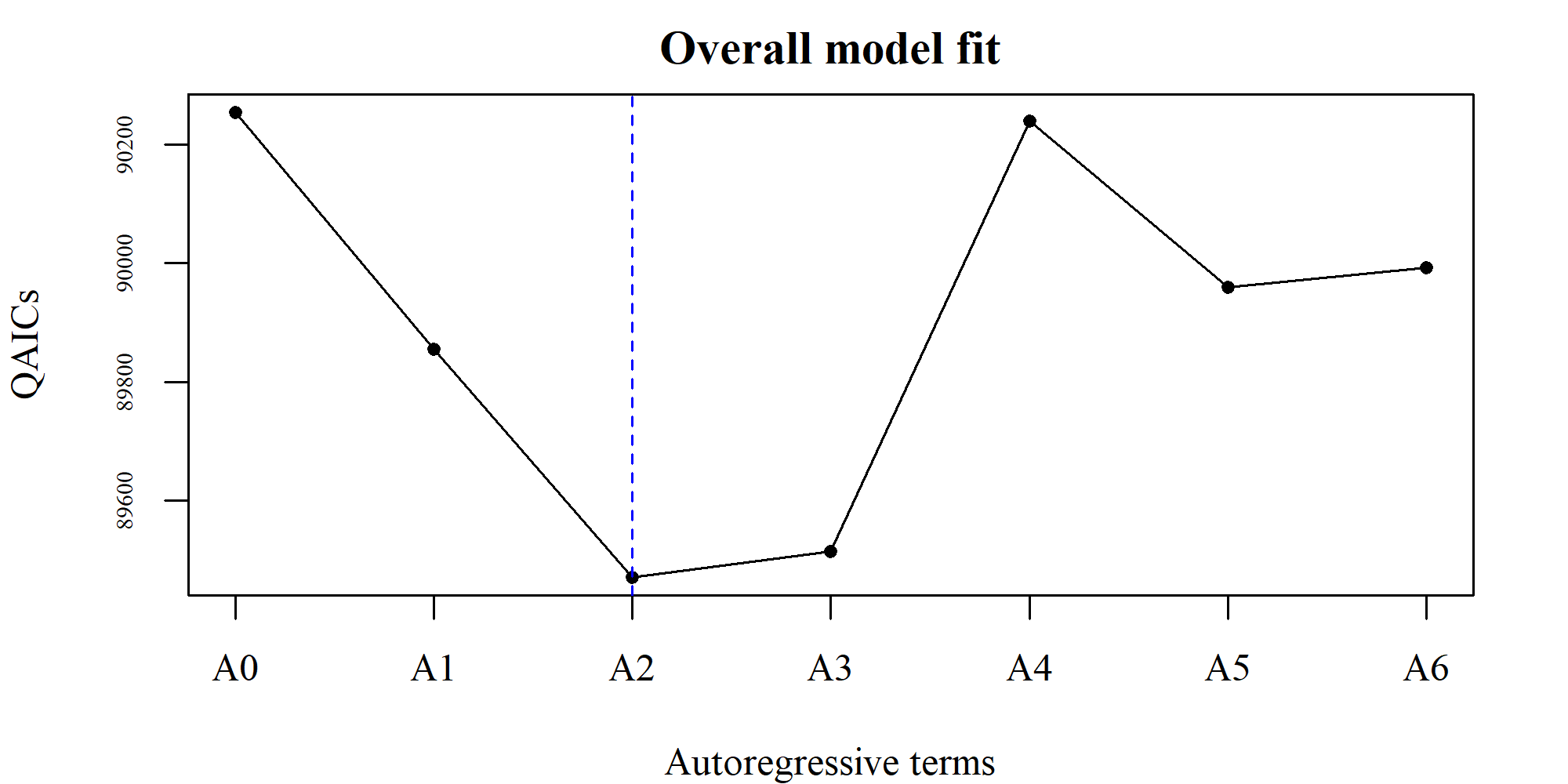


Fig H. The overall model fit of different autoregressive term settings


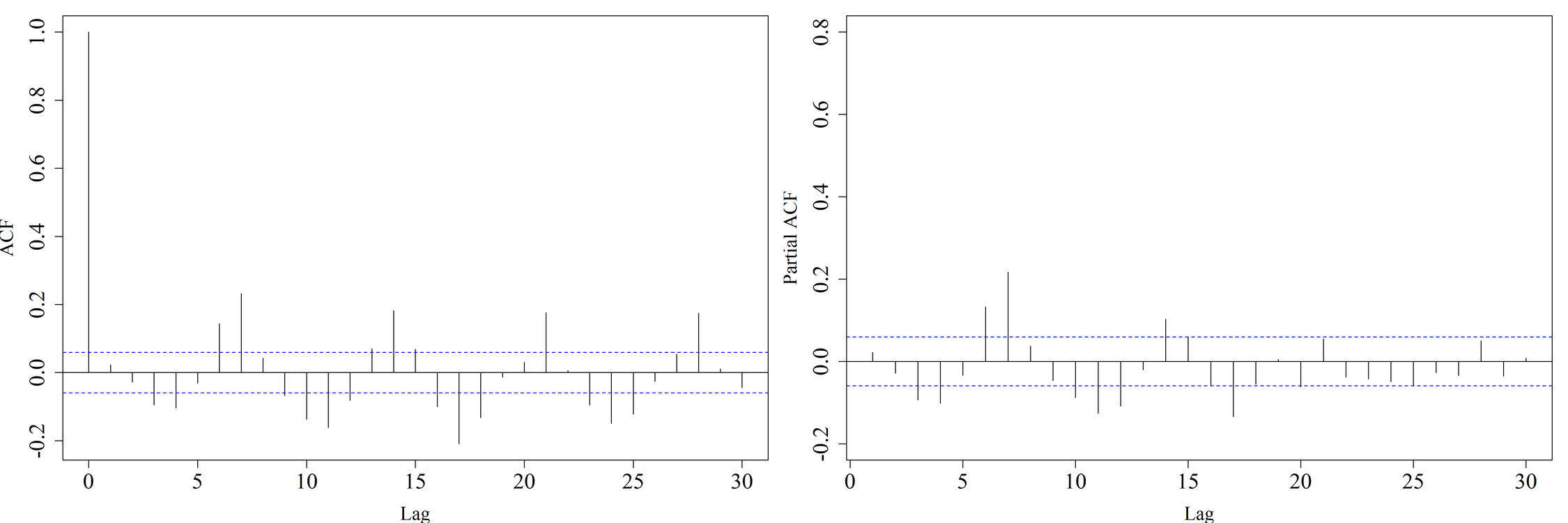


Fig I. The ACF and PACF analysis on the residuals of the HFMD cases after controlling autoregressive term

1. *The choice of lag days of air pollutants*

Considering that HFMD always has two weeks infection period, we chose 0-14 days as the lag intervals for PM_10_, SO_2_, NO_2_ and CO.

1. *The choice of the* *dfs of splines for the lag structure of air pollutants.*

For the choice of the *df*s of splines for the lag structure of air pollutants, we set up natural cubic splines with 3-6 *df*s, respectively. For all air pollutants in our study, the model with the natural cubic splines with 3 *df* had minimize QAICs. Therefore, we chose the natural cubic splines with 3 *df* for the lag structure of air pollutants.


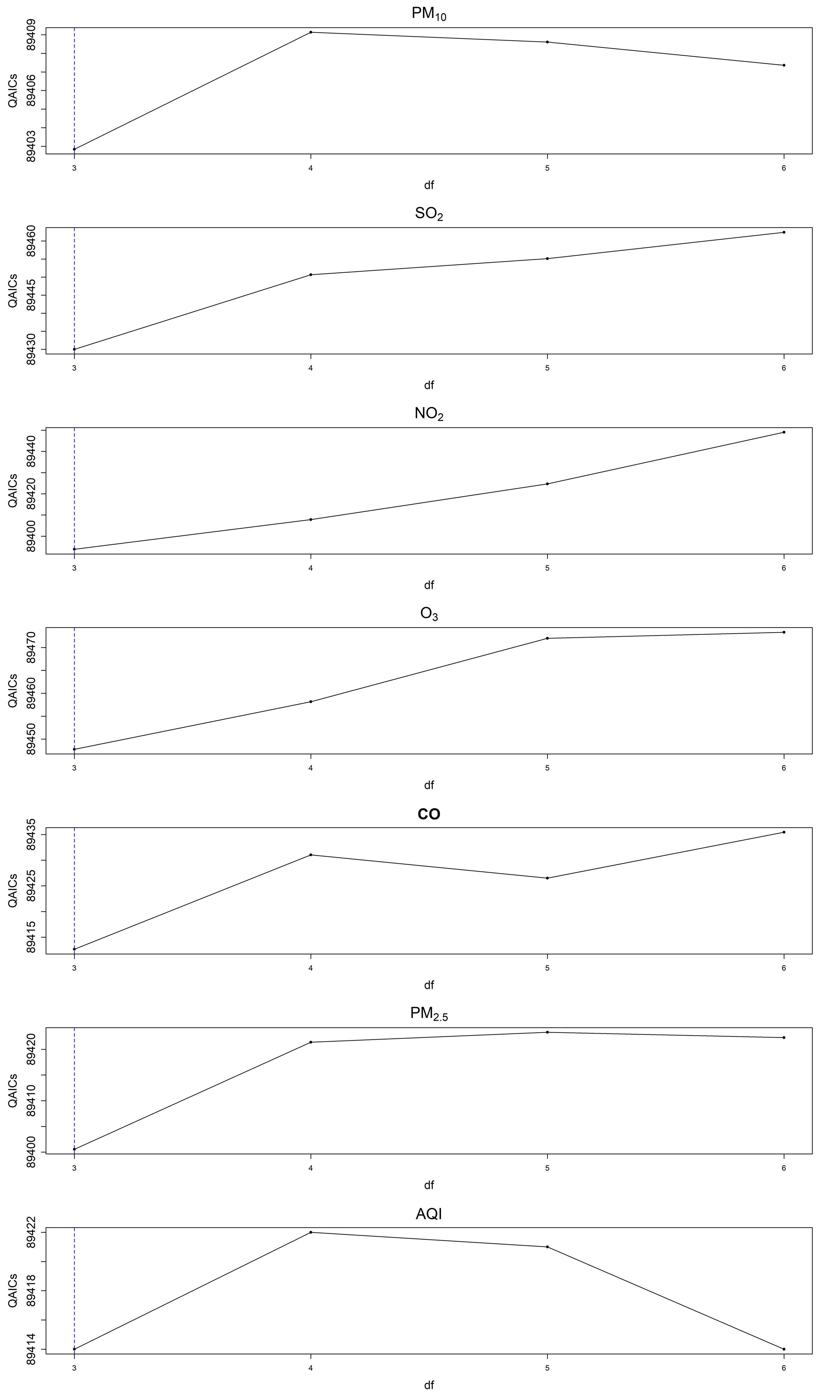


Fig J. The QAICs of different *df*s of splines for lag-response structure of air pollutants

1. *The choice of the knots of splines for the exposure-response structure of air pollutants.*

For the choice of the knots of splines for the exposure-response structure of air pollutants, we set up natural cubic splines with 2-4 knots, respectively. For each air pollutant, we calculated arithmetic mean concentration of 17 cities for every day. Then we chose the values of knots as Table D. Table E showed the overall model fits of each pollutant. The models with the natural cubic splines with 3 knots had minimize QAICs for PM_2.5_ and with 2 knots had minimize QAICs for other pollutants. Therefore, we chose the natural cubic splines with 3 knots for PM_2.5_ and 2 knots for the exposure -response structure of air pollutants.

Table D. The values of different knots of splines for the exposure -response structure of air pollutants

| Notation | *knots*= 2  (33^th^, 66^th^) | *knots*= 3  (25^th^, 50^th^, 75^th^) | *knots*= 4  (20^th^, 40^th^, 60^th^, 80^th^) |
| --- | --- | --- | --- |
| PM_10_ (μg/m^3^) | (54.3, 86.0) | (47.8, 67.9, 101.0) | (44.9, 60.1, 77.5, 111.2) |
| SO_2_ (μg/m^3^) | (11.9, 15.7) | (11.1, 13.7, 16.9) | (10.6, 12.8, 14.8, 17.8) |
| NO_2_ (μg/m^3^) | (25.4, 33.5) | (23.8, 29.2, 36.4) | (22.6, 26.9, 31.8, 38.2) |
| O_3_ (μg/m^3^) | (45.0, 70.5) | (39.3, 57.8, 78.2) | (36.1, 50.0, 66.2, 83.8) |
| CO (mg/m^3^) | (0.7, 0.9) | (0.7, 0.8, 1.0) | (0.7, 0.8, 0.9, 1.1) |
| PM_2.5_(μg/m^3^) | (31.8, 52.6) | (27.9, 40.8, 64.3) | (25.3, 35.5, 48.1, 70.2) |
| AQI | (58.4, 82.5) | (53.5, 68.7, 93.5) | (50.5, 62.2, 76.3, 99.8) |

Table E. The QAICs of different knots of splines for the exposure -response structure of air pollutants

| Notation | *knots*= 2 | *knots*= 3 | *knots*= 4 |
| --- | --- | --- | --- |
| PM_10_ | 89,353.0 | 89,370.9 | 89,437.0 |
| SO_2_ | 89,409.8 | 89,423.4 | 89,454.4 |
| NO_2_ | 89,454.1 | 89,506.6 | 89,528.3 |
| O_3_ | 89,472.0 | 89,520.6 | 89,534.6 |
| CO | 89,555.7 | 89,607.1 | 89,615.6 |
| PM_2.5_ | 89,598.9 | 89,598.8 | 89,643.8 |
| AQI | 89,358.4 | 89,407.9 | 89,458.0 |
